# The Dynamic Transcriptional Cell Atlas of Testis Development during Human Puberty

**DOI:** 10.1016/j.stem.2019.12.005

**Published:** 2020-02-06

**Authors:** Jingtao Guo, Xichen Nie, Maria Giebler, Hana Mlcochova, Yueqi Wang, Edward J. Grow, DonorConnect, Robin Kim, Melissa Tharmalingam, Gabriele Matilionyte, Cecilia Lindskog, Douglas T. Carrell, Rod T. Mitchell, Anne Goriely, James M. Hotaling, Bradley R. Cairns

**Affiliations:** 1 Howard Hughes Medical Institute, Department of Oncological Sciences and Huntsman Cancer Institute, University of Utah School of Medicine, Salt Lake City, UT 84112, USA; 2 The Andrology Laboratory, Department of Surgery (Andrology/Urology), Center for Reconstructive Urology and Men’s Health, University of Utah Health Sciences Center, Salt Lake City, UT 84112, USA; 3 Radcliffe Department of Medicine, MRC Weatherall Institute of Molecular Medicine, University of Oxford, Oxford OX39DS, UK; 4 Department of Computer Science, Columbia University, New York, NY 10027, USA; 5 DonorConnect, Murray, UT 84107, USA; 6 Section of Transplantation, Department of Surgery, University of Utah School of Medicine, Salt Lake City, UT 84132, USA; 7 MRC Centre for Reproductive Health, The Queen’s Medical Research Institute, The University of Edinburgh, Edinburgh EH16 4TJ, UK; 8 Royal Hospital for Children and Young People, Edinburgh EH91LF, UK; 9 Department of Immunology, Genetics and Pathology, Science for Life Laboratory, Uppsala University, Uppsala 751 85, Sweden

**Keywords:** single cell sequencing, puberty, spermatogonial stem cell, state 0, Sertoli cell, Leydig cell, peritubular myoid cell, transfemale, testosterone, testis maturation

## Abstract

The human testis undergoes dramatic developmental and structural changes during puberty, including proliferation and maturation of somatic niche cells, and the onset of spermatogenesis. To characterize this understudied process, we profiled and analyzed single-cell transcriptomes of ∼10,000 testicular cells from four boys spanning puberty and compared them to those of infants and adults. During puberty, undifferentiated spermatogonia sequentially expand and differentiate prior to the initiation of gametogenesis. Notably, we identify a common pre-pubertal progenitor for Leydig and myoid cells and delineate candidate factors controlling pubertal differentiation. Furthermore, pre-pubertal Sertoli cells exhibit two distinct transcriptional states differing in metabolic profiles before converging to an alternative single mature population during puberty. Roles for testosterone in Sertoli cell maturation, antimicrobial peptide secretion, and spermatogonial differentiation are further highlighted through single-cell analysis of testosterone-suppressed transfemale testes. Taken together, our transcriptional atlas of the developing human testis provides multiple insights into developmental changes and key factors accompanying male puberty.

## Highlights

• A transcriptional single-cell atlas of the developing testes during human puberty
• Distinctive phases of germ cell differentiation occur during puberty
• Identification of a common progenitor for Leydig and myoid cells prior to puberty
• Partial reversal of Sertoli and germ cell maturation upon testosterone suppression

## Graphical Abstract

**Figure:**
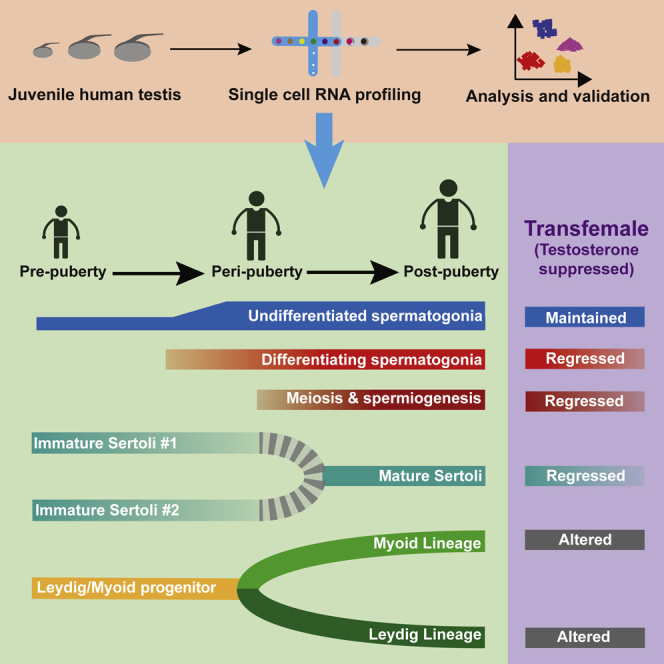

## Teaser

Guo et al. provide a transcriptional cell atlas of the developing human testis during puberty, revealing dramatic developmental changes in both germ and somatic niche cell lineages. Furthermore, germ cells and Sertoli cells from testosterone-suppressed transfemale testes display partial developmental reversal, revealing critical roles for testosterone in maintaining testis maturation.

## Introduction

Human male puberty involves major changes in testis physiology, a large increase in testicular volume, and complex hormonal and molecular modulation, to accomplish both somatic cell proliferation/maturation and the initiation of spermatogenesis (Koskenniemi et al., 2017, Sharpe et al., 2003). Puberty is initiated by re-activation of the hypothalamo-pituitary-gonadal axis following a period of relative quiescence during childhood (Plant, 2015). This hormonal control requires hypothalamic gonadotrophin-releasing hormone (GnRH) stimulating the release of gonadotrophins, luteinizing hormone (LH), and follicle stimulating hormone (FSH). LH is responsible for stimulating Leydig cells to produce testosterone (T), while FSH stimulates Sertoli cells to support spermatogenesis (Herbison, 2016, Plant, 2015).

To date, our understanding of puberty derives mostly from physiological approaches in humans and primates complemented by extensive molecular and genetic approaches in rodents, which includes the use of sophisticated lineage-tracing and transgenic technologies. However, fundamental differences exist between human and rodent with respect to hormonal control of puberty and onset of spermatogenesis (Tharmalingam et al., 2018). In mice, type B spermatogonia begin to develop at day 8 (d8) resulting in a synchronous “first wave” of spermatogenesis (Bellvé et al., 1977), during which pro-spermatogonia undergo differentiation and synchronously generate both self-renewing and differentiating spermatogonia (Kluin and de Rooij, 1981, Yoshida et al., 2006). This results in the first round of murine sperm production at around d35 (Vergouwen et al., 1993). Subsequently, self-renewing spermatogonia, residing in the niche, commence differentiation and produce successive and continuous waves of gametogenesis from 2–3 weeks after birth (Kanatsu-Shinohara and Shinohara, 2013). However, humans lack the equivalent of this first wave of spermatogenesis and instead are believed to maintain spermatogonia in an undifferentiated (though largely uncharacterized) state prior to the initiation of puberty (Paniagua and Nistal, 1984).

In humans, puberty typically begins between 10 and 13 years after birth. However, low levels of incomplete spermatogenesis is observed in portions of the testis in juveniles prior to puberty, though mature sperm are usually not present prior to age 10–11, and meiotic cells typically undergo apoptosis during peri-pubertal phases (Chemes, 2001) (note: we define “juvenile” as the developmental period after the infant stage but prior to full adult sexual maturity [typically ages from 1–14]). Morphologically, the pre-pubertal testis resembles that of the early postnatal mouse, with undifferentiated spermatogonia embedded in small “cords” of Sertoli cells, that lack a defined tubular lamina or lumen. During puberty, formation of the basal lamina and lumen creates structured seminiferous tubules (Paniagua and Nistal, 1984), which involves the coordinated development of myoid cells (contributing to the formation of the basal lamina), Leydig cells (responsible for hormonal signaling), and Sertoli cells with multiple functions within the tubule—and which together define the main niche components. However, our understanding of this crucial developmental period is limited, and much remains to be characterized at the molecular and genome-wide level, including the mechanisms that maintain human spermatogonial stem cells (SSCs) in a slowly self-renewing and undifferentiated state during childhood and the processes responsible for initiation and progression to spermatogenesis at puberty.

Single-cell RNA sequencing (scRNA-seq) approaches provide unique and powerful tools to examine human testis development, enabling the simultaneous examination of thousands of individual cells, faithfully representing the constituents of entire organs, without the need for prior sorting or enrichment procedures (Birnbaum, 2018, Guo et al., 2018, Hermann et al., 2018, Sohni et al., 2019, Wang et al., 2018). The lack of spatial information obtained from this approach can be alleviated by using orthogonal validation approaches such as co-localization of specific proteins in fixed tissues (Guo et al., 2018). Here, we profiled ∼10,000 single-cell transcriptomes from whole-testes of four juvenile males (7, 11, 13, and 14 years old) and compared these data to our previously published infant (1 year old) and adult (25 years old) datasets (Guo et al., 2018). Our study uncovers dramatic changes in germline stem cells and heretofore unappreciated, complex modulations of the somatic niche revealing candidate factors and pathways that regulate somatic cell development during puberty. Certain changes in the niche, such as T production, are accompanied by changes in germ cell states—indicating communication between the germline and niche. To explore the role of T on germline and niche development and function, we analyzed the transcriptome of testes from T-suppressed adult transfemales (that retained spermatogonia), which revealed roles for T in promoting and/or supporting testis development during puberty. Taken together, our datasets and comparative analyses provide multiple insights into the development of SSCs and their niche during puberty in humans, while also providing a valuable data resource (https://humantestisatlas.shinyapps.io/humantestisatlas1/) for the research community.

## Results

### Single-Cell Transcriptomes of Human Juvenile Testes

**Figure 1:**
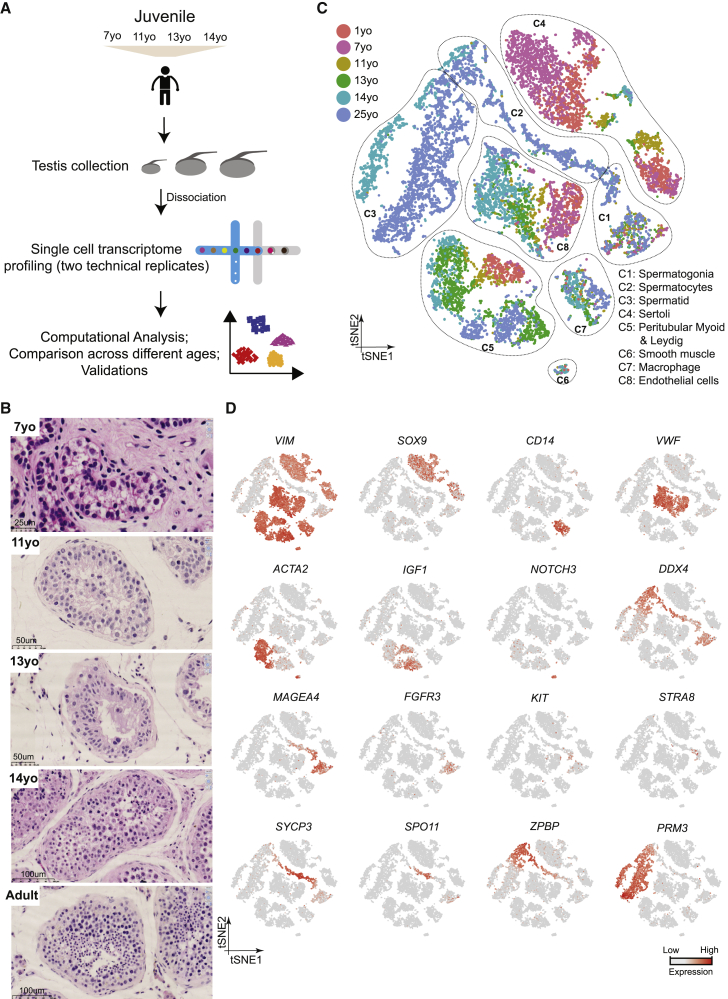
Single-Cell Transcriptome Profiling of the Human Juvenile Testis (A) Schematic illustration of the experimental workflow. (B) H&E (hematoxylin and eosin) staining of the different juvenile testes analyzed in this study illustrates the typical macroscopic testicular changes in morphology observed during puberty. (C) tSNE and clustering analysis of single-cell transcriptome data from juvenile human testes (n = 7,680), combined with previous datasets of infant and adult scRNA-seq (Guo et al., 2018). Each dot represents a single cell and is colored according to its donor of origin. tSNE, t-distributed stochastic neighbor embedding. (D) Expression patterns of selected markers projected on the tSNE plot (Figure 1C). For each cell cluster, one cell marker is shown in the main figure accompanied by a gallery of further markers in Figures S1D and S1E. The two top rows show somatic/niche cell markers; representative germ cell markers are shown in the two bottom rows. See also Figure S1 and Table S1.

We collected whole-testes through a rapid autopsy program from 4 juvenile donors aged 7, 11, 13, and 14 years (Figure 1A), with Johnsen scores (Johnsen, 1970) at 3, 4, 4, and 7, respectively. Consistent with prior observations (Paniagua and Nistal, 1984), our histological examination revealed major changes in morphology and composition along this timeline (Figure 1B): in the 7-year-old sample, small testis cords lacking an apparent lamina or lumen were observed, forming structures reminiscent of those seen in fetal/early postnatal mouse testes. A tubular structure became progressively apparent in the 11-year-old sample, while a clear defined lamina and lumen were observed across the tubules of the 13- and 14-year-old samples (Figure 1B). To characterize the molecular features accompanying this developmental progression, we isolated single cells from these testicular tissues and performed scRNA-seq using the 10x Genomics platform. For each donor, two separate technical replicates were performed, resulting in eight datasets. From a total of ∼10,000 cells, 7,675 passed standard quality-control (QC) dataset filters and were retained for downstream analysis (see STAR Methods for details). We obtained ∼200K reads/cell, which enabled the analysis of ∼1,500–2,000 genes/cell (Figure S1A). The sequencing saturation rate was ∼85%, and technical replicates were highly similar (r > 0.95) (Figures S1A and S1B).

To describe pubertal development and to enable comparison to other time points, we combined the juvenile datasets with our previously published adult and infant datasets, yielding a total of 13,797 single cells (after QC filtering). We first performed t-distributed stochastic neighbor embedding (tSNE) analysis on the combined datasets using the Seurat package (Butler et al., 2018; Figure 1C), which yielded eight major clusters that were subsequently annotated using known gene markers (Figure 1D; Figures S1D and S1E). Clusters 1–3 are germ cells, with C1 representing spermatogonia (*UTF1*^+^, *FGFR3*^+^), C2 consisting of spermatocytes (*SYCP3*^+^), and C3 identifying post-meiotic spermatids (*PRM3*^+^). Clusters 4–8 correspond to a heterogenous mixture of Sertoli cells (C4; *SOX9*^+^), Leydig and myoid-like cells (C5; *IGF1*^+^ and/or *ACTA2*^+^), smooth muscle (C6; *NOTCH3*^+^), macrophages (C7: *CD14*^+^), and endothelial cells (C8; *VWF*^+^), respectively (see Figures S1D and S1E for additional markers).

### Distinct Phases of Spermatogonial Proliferation and Differentiation during Human Puberty

**Figure 2:**
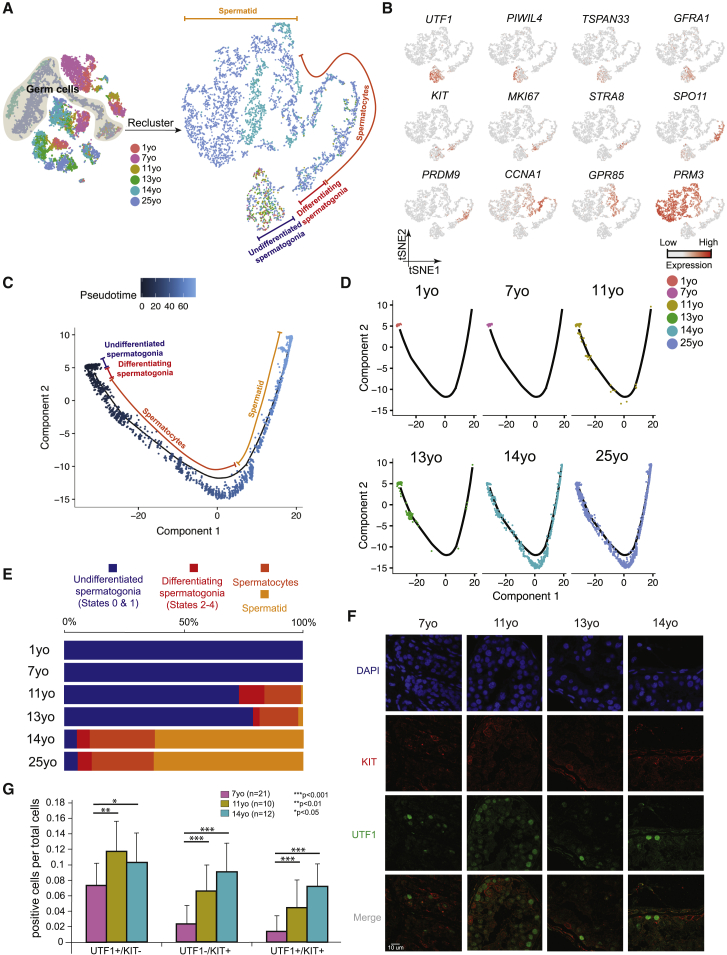
Distinct Phases of Spermatogonial Proliferation and Differentiation during Human Puberty (A) Focused analysis (tSNE and clustering) of the germ cells (clusters C1, C2, and C3 from Figure 1C) reveals developmental progression of spermatogenesis during puberty. Cells are colored based on the ages/donors of origin. (B) Expression patterns of known spermatogenic markers projected onto the tSNE plot from Figure 2A. (C) Pseudotime trajectory (Monocle analysis) of the germ cells. Cells are colored based according to the predicted pseudotime. (D) Deconvolution of the Monocle pseudotime plot according to ages/donors of origin. (E) Relative proportion of the single cells at different spermatogenic stages in the samples analyzed. (F) Protein co-immunofluorescence for two spermatogonial cell markers: UTF1 (States 0–1) and KIT (States 2–4) in the 4 analyzed samples. See Figure S2D for a wider field of view. (G) Quantification of UTF1^+^ and/or KIT^+^ spermatogonia at different ages. The data shown are means ± SD of independent tubules. The p value was calculated via Student’s t test. See also Figure S2.

We examined changes in germ cell composition during puberty by analyzing the germ cells, following specific reclustering of initial clusters 1–3 (Figure 2A). Expression patterns of key markers and comparisons to prior work enable assignment of germ cells into four broad developmental stages: slowly self-renewing and undifferentiated spermatogonia (referred to as States 0–1 in [Guo et al., 2017, Guo et al., 2018]) marked by *UTF1*^+^ and largely *MKI67*^*–*^, differentiating spermatogonia (States 2–4) marked by *KIT*^+^ and largely *MKI67*^+^ (note: State 4 is preparing to enter meiosis and lacks *MKI67*), spermatocytes (from *STRA8*^*+*^ to *GPR85*^+^), and spermatids (*PRM3*^+^) (Figures 2A and 2B). An orthogonal pseudotime analysis using the Monocle package (Qiu et al., 2017) further supports this pseudotime trajectory (Figures 2C and 2D; Figures S2A and S2B). Germ cells were then parsed out to examine their relative composition at different phases of puberty (Figures 2D and 2E). Interestingly, germ cells from both the 1- and 7-year-old samples consisted of only undifferentiated spermatogonia (corresponding to States 0–1) (Figures 2A, 2D, and 2E) that expressed *UTF1* and other early germline stem cell markers (Figure 2B; Figures S2A and S2B). In the 11-year-old sample, while a high proportion of cells were still States 0–1 spermatogonia, differentiating spermatogonia and meiotic cells also began to emerge (Figures 2D and 2E). Notably, in the infant and 7-year-old samples, spermatogonia were relatively rare (∼3%–4% of total testicular cells), whereas in the 11-year-old and older samples, the relative proportion of spermatogonia increased considerably to represent ∼10%–15% of total testicular cells (Figure S1C), consistent with a spermatogonial amplification and proliferative phase prior to a differentiation phase. The 13-year-old sample largely resembled the 11-year-old sample (likely reflecting the known age differences in puberty onset) though post-meiotic cells increased in proportion, indicating a more robust commitment to meiosis. Last, germ cell composition in the 14-year-old sample resembled the adult, indicating nearly full spermatogenesis (Figure 2E).

Next, we performed immunofluorescence (IF) to confirm our scRNA-seq findings (Figures 2F and 2G; Figures S2C and S2D). First, we observed UTF1^+^ undifferentiated spermatogonia (State 0–1) at all ages analyzed. In contrast, proliferative and differentiating spermatogonia (States 2–3) display strong increases in multiple proliferative markers (e.g., cyclins, CDKs, *TOP2A*, MKI67, KIT [Figure S2E]) and MKI67^+^ spermatogonia become apparent only from 11 years old onward, and significant levels of the meiotic marker SYCP3 were found only at/after 14 years old (Figure S2C). Co-staining for UTF1 and KIT confirmed the gradual and steady increase in KIT^+^ spermatogonia during juvenile development (Figures 2F and 2G), indicative of progressive commitment to spermatogonial differentiation. Taken together, our transcriptomic and protein data combined with prior immunohistochemistry (IHC) studies (Paniagua and Nistal, 1984) point to three separate phases between infancy and puberty: (1) a phase where undifferentiated spermatogonia are quiescent or slowly self-renew, (2) a transient period of spermatogonial proliferation (mitosis) with limited and incomplete spermatogonial differentiation, occurring early in puberty, which gradually transitions to (3) the establishment of steady and balanced spermatogonial self-renewal and differentiation, alongside commitment to full gametogenesis.

### Gene Expression Dynamics during Sertoli Cell Maturation

**Figure 3:**
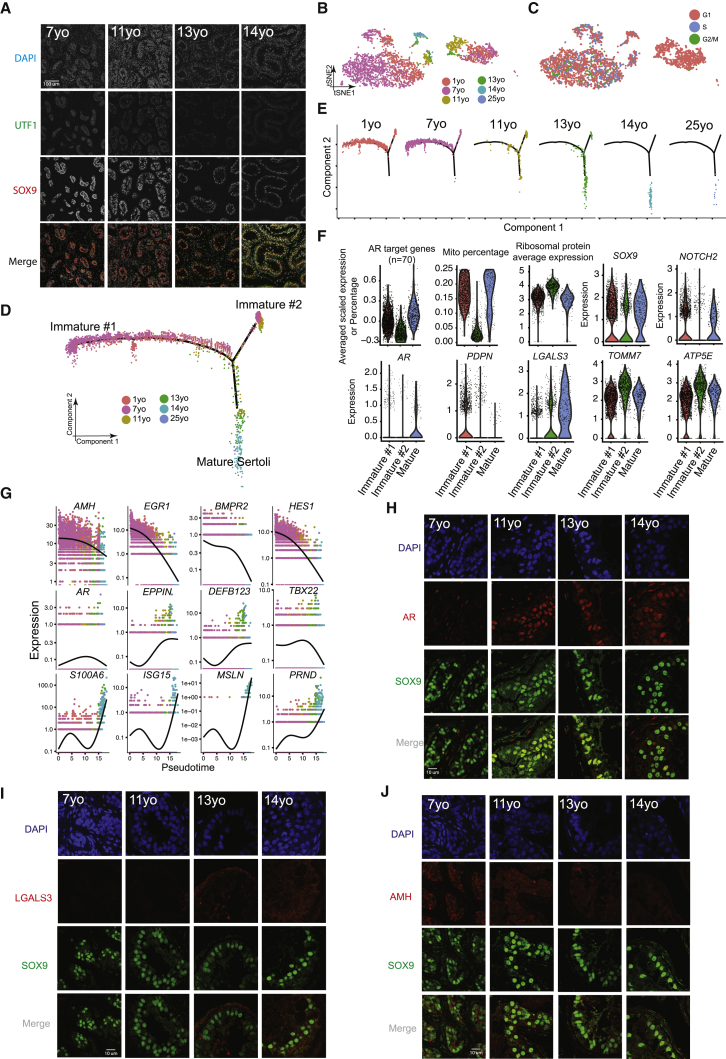
Identification of Two Sertoli States in the Pre-pubertal Testis (A) Immunolocalization of germ cells and Sertoli cells in the analyzed samples (7–14 years old) illustrated by co- staining with UTF1 and SOX9. (B) Focused analysis (tSNE and reclustering) of Sertoli cells (cluster C4 from Figure 1C), with cells colored by ages/donors. (C) Focused analysis (tSNE and reclustering) of Sertoli cells, with cells colored according to their cell-cycle phase (G1/S/G2). (D) Pseudotime trajectory (via Monocle) of Sertoli cells revealed two distinct early (immature) cellular states that progressively converge along the pseudotime. (E) Deconvolution of the Monocle pseudotime plot according to ages/donors of origin. (F) Selected key genes/programs showing differential expression in the distinct Sertoli states. (G) Expression patterns of representative dynamic genes during Sertoli cell maturation, as predicted by pseudotime. (H) Immunofluorescent co-staining for SOX9 and AR at different ages (7–14 years old) shows an increase in AR expression during juvenile development. (I) Immunofluorescent co-staining for SOX9 and LGALS3 at different ages (7–14 years old) supports the progressive maturation of the Sertoli cells shown by pseudotime trajectory. (J) Immunofluorescent co-staining for SOX9 and AMH at different ages (7–14 years old) supports the gradual maturation of Sertoli cells over time shown by pseudotime trajectory. See also Figures S3–S5 and Table S2.

We next explored Sertoli cell development during puberty. As previewed above, in the pre-pubertal samples (7 years old), staining of UTF1 and SOX9 revealed cord-like structures consisting of intermingled spermatogonia and Sertoli cells (Figure 3A). On initiation of puberty (e.g., 11- and 13-year-old samples), Sertoli cells and spermatogonia align at the basement membrane of the tubules and a lumen begins to form—a pattern that developed further in the 14-year-old sample (Figure 3A). To characterize this morphological re-organization, we parsed out and examined the Sertoli cell populations (cluster 4 in Figure 1C). Here, we note that mature Sertoli cells are typically larger than the preferred size for capture in the 10x Genomics system, likely explaining the low proportion of Sertoli cells at older ages. Hence, although we obtained sufficient cell numbers to define Sertoli cell transcriptional signatures, this caveat prevents quantitation of changes in total Sertoli cell numbers during development. Our clustering analysis defined two large Sertoli clusters flanking two smaller clusters (Figures 3B and 3C). Notably, cell-cycle analysis (see STAR Methods) reveals that the large right cluster (Figures 3B and 3C) contains cells from all juvenile samples and displays a G1 phase-bias, while the left cluster is mainly composed of cells from the younger samples (1–7 years old) at different cell-cycle phases. Interestingly, Monocle pseudotime analysis also partitioned the 1- and 7-year-old Sertoli cells into two distinct states, termed Immature #1 and #2 (Figures 3D and 3E). Remarkably, these two states merge into one path that lead to mature Sertoli cells, which start emerging in samples from 11 years old onward (Figures 3D and 3E).

We observed multiple features of Immature #1 and mature Sertoli cells that distinguish them from Immature #2 cells, including high AR (Androgen Receptor) target gene expression (defined by a set of ∼70 known AR-dependent genes) (Bolton et al., 2007), high mitochondrial transcription, and lower expression of ribosomal protein genes (Figure 3F). Gene Ontology (GO) analysis revealed enrichment for terms such as nucleus and transcription in Immature #1 (Figure S3H). IF co-staining for AR and SOX9 confirms this sequence of events, by showing gradual AR upregulation and co-localization in Sertoli cells as puberty proceeds (Figure 3H). Immature #2 cells have the converse properties of Immature #1 described above and are enriched in GO terms for translation and ribosome function (Figure S3H). They exhibit higher transcription of ribosomal protein genes, mitochondrial ATPases (e.g., *ATP5E*), and mitochondrial membrane proteins (e.g., *TOMM7*) and contain a higher fraction of cells in the G1 phase of the cell cycle (Figures 3F and S3E). Taken together, these two immature Sertoli cell states differ markedly in their metabolic/mitochondrial, translational, and cell-cycle properties (as indicated by transcriptional signatures).

Analysis of Sertoli maturation via pseudotime and differential gene expression revealed dynamic expression of ∼1,000 genes (Figure S3F) that differ between the immature and mature stages. As expected, genes associated with cytoskeleton, cell morphogenesis, and extracellular matrix were upregulated during Sertoli maturation (Griswold, 2018; Figure S3F). Genes encoding antimicrobial innate immunity peptides (including the defensin class of peptides) and immunity-related genes (e.g., *ISG15*) were also upregulated during puberty (Figures 3F, 3G, and S3G), supporting a role for Sertoli cells in protecting the testis from infection (Com et al., 2003), especially after sexual maturation. To confirm this, we performed IF/IHC staining and observed downregulation of the immature Sertoli marker AMH during puberty (Figures 3J and S3I), and upregulation of the immune peptides LGALS3 and S100A6 in Sertoli cells in the 14-year-old sample (Figures 3I and S3J). Interestingly, immunostaining of an Immature #1 marker, PDPN (Figure 3F), showed spatial separation in the 11-year-old testis (Figure S3D), suggesting that the maturation of Sertoli cells in different tubules is asynchronous and proceeds gradually. Taken together, our data describe complex developmental progression in Sertoli cell states late in puberty that involve morphogenesis, metabolism, and innate immunity.

### A Common Progenitor for Leydig and Peritubular Myoid Cells

**Figure 4:**
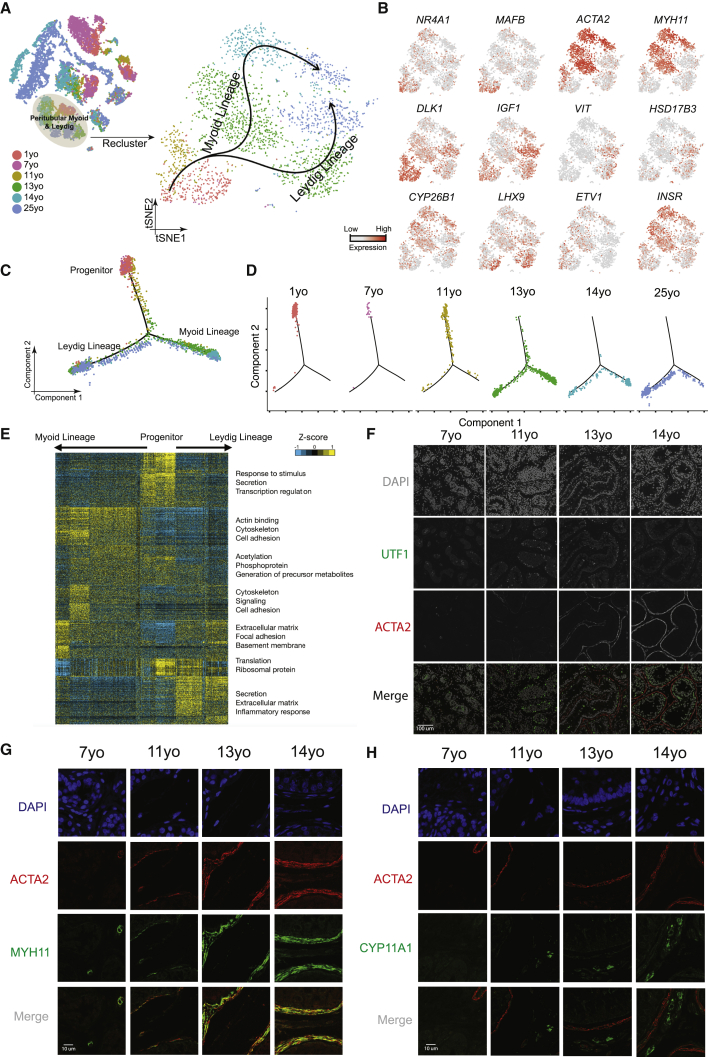
A Common Progenitor for Leydig and Peritubular Myoid Cells (A) Focused analysis (tSNE and clustering) of the Leydig and peritubular myoid cells (cluster C5 from Figure 1C), with cells colored according to ages/donors of origin. The predicted developmental lineages are represented by the arrows. (B) Expression patterns of key representative markers projected onto the tSNE plot from Figure 4A. (C) Pseudotime trajectory (via Monocle) of the C5 cluster predicts a common early pre-pubertal progenitor state and two distinct developmental trajectories for the Leydig and myoid lineages during puberty. (D) Deconvolution of the Monocle pseudotime plot according to the ages/donors of origin. (E) K-means clustering of genes exhibiting differential expression (n = 1,005) along the Leydig and myoid cell lineages. Each row represents a gene, and each column represents a single cell, with columns/cells placed in pseudotime order defined in Figure 4C. Differential gene expression levels utilize a *Z* score as defined by the color key; associated GO terms (using DAVID v6.7) are given on the right of the corresponding gene clusters. (F) Protein co-immunofluorescence for UTF1 and the peritubular myoid cell marker ACTA2 in the analyzed samples (7–14 years old) revealed that the myoid lineage (ACTA2^+^) is progressively specified during puberty. (G) Protein co-immunofluorescence for two known myoid cell markers, ACTA2 and MYH11, at different ages (7–14 years old). (H) Immunofluorescent co-staining for ACTA2 and CYP11A1 (Leydig cell marker) shows the progressive expression of them during juvenile development. See also Figures S4 and S5 and Table S3.

Interestingly, the clustering analysis had initially placed Leydig and myoid cells together into a single large cluster (C5; Figure 1C), prompting further exploration. Reclustering of C5 revealed that cells from the 11-year-old and younger samples largely overlapped and shared the expression of known markers of fetal Leydig and myoid precursors in the mouse (e.g., *MAFB* and *NR4A1*; Figures 4A and 4B; DeFalco et al., 2011, Wen et al., 2016), as well as heterogeneous co-expression of markers typically associated with both mature Leydig and myoid cells (e.g., *DLK1* and *ACTA2*, respectively) (Guo et al., 2018). However, in samples from 13 years old to adult, we observed a clear separation of Leydig (marked by *DLK1* and *IGF1*) and myoid cells (marked by *ACTA2* and *MYH11*). In keeping, Monocle pseudotime analysis revealed that cells from pre-puberty (1–7 years old) tightly cluster together, and the trajectory bifurcates into two clusters as puberty proceeds (Figures 4C and 4D; Figures S4C and S4D). Thus, both modes of computational analysis suggest a common expression program defining a bipotential progenitor for Leydig and myoid cells prior to puberty.

We next identified lineage-specific genes and programs, yielding ∼1,000 differentially expressed genes (Figure 4E). Early precursors expressed particular genes associated with transcription (e.g., *MAFB* and *NR4A1*); as cells progress toward the myoid lineage, cytoskeleton- and cell adhesion-related genes become expressed, consistent with myoid cells forming their distinctive peritubular structure (Maekawa et al., 1996). In contrast, cells progressing along the Leydig lineage express genes involved in secretion, consistent with the known steroidogenic function of these cells (Chen et al., 2009). IF stainings revealed that ACTA2 and MYH11 signals were low or absent in the tubule lamina in the 7- and 11-year-old samples, which display a layered ring structure around the tubules from 13 years old and onward (Figures 4F and 4G). Likewise, protein expression of CYP11A1, a marker for Leydig cell steroidogenesis, progressively appears as puberty proceeds (Figure 4H).

### Orthogonal Validation using Additional Juvenile Testis Samples

To further validate our results, we performed IHC on five additional infant-juvenile samples (1, 2, 4, 11, and 14 years old) (Figures S4E and S4F). In keeping with the results above, we observed progressive upregulation of AR expression during puberty, concomitant with gradual AMH downregulation as Sertoli cells mature (Figure S4E). Furthermore, in these independent samples the expression of CYP11A1 was evident only in the 14-year-old sample, indicating complete maturation of adult Leydig cells. Likewise, the myoid marker, ACTA2, displays clear laminar expression only later in puberty (14 years old, Figure S4F). These stainings provide additional confirmation for the generality of our genomics findings and validation of the 4 analyzed testis samples.

### Signaling Pathways Regulating Testis Development during Puberty

To explore germ cell-niche and niche-niche interactions during puberty, we examined signaling factor relationships (Figure S5). We observed dynamic and cell-type-specific expression of genes encoding ligands, inhibitors, receptors, and gene targets from multiple signaling pathways, including retinoic acid (RA), activin/inhibin, NOTCH, GDNF, FGF, and WNT (Figures S5B–S5H). This genome-wide view confirmed known relationships, previously documented in either humans or mice, while other observations provide useful cues regarding temporal and molecular relationships during puberty. For example, as *STRA8* expression was absent from the 7- to 11-year-old samples (Figure S5B), RA is unlikely to be involved in the transition from undifferentiated to differentiating spermatogonia during human puberty, in contrast with findings in pre-pubertal mice (Velte et al., 2019). Furthermore, our data support Activin and BMP pathways as candidate key players during puberty: we observe that Sertoli cells downregulate *INHA* as they mature, while Leydig cells express high levels of *INHBA* after puberty, leading to increased Activin and lower Inhibin activity after puberty (Figure S5C). IHC staining confirmed that INHA protein is downregulated as puberty proceeds (Figure S5D). Interestingly, activin receptors (*ACVR1B*, *BMPR1B*, and *ACVR2B*) are expressed in spermatogonia, whereas key inhibitors of Activin signaling (*FST*, *BAMBI*, and *NOG*) are specifically expressed in the undifferentiated spermatogonia (States 0 and 1). Thus, the Activin pathway appears selectively inhibited in slowly self-renewing and undifferentiated spermatogonia but is active in proliferating and differentiating spermatogonia, warranting further exploration for functional roles during spermatogonial differentiation.

### Single-Cell Profiling of Testes from T-Suppressed Transfemales

**Figure 5:**
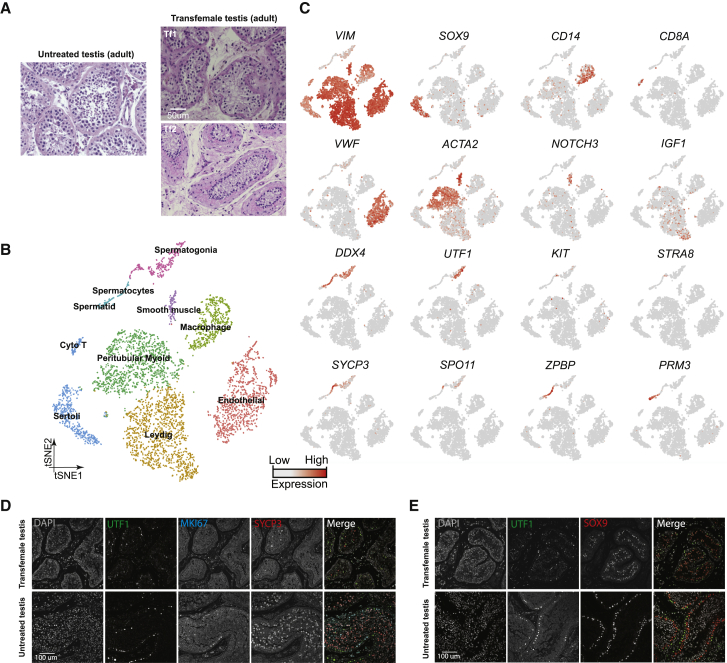
Single-Cell Transcriptome Profiling of Testes from T-Suppressed Transfemales (A) H&E staining of the adult untreated (25 years old) and T-suppressed (Tf1 and Tf2) testicular sections. (B) tSNE and clustering analysis of single-cell transcriptome data from two transfemale testes (n = 5,179). (C) Expression patterns of selected markers projected on the tSNE plot. Top two rows are somatic/niche cell markers; bottom two rows are representative germ cell markers. (D) Examination of germ cell compositions in T-suppressed (Tf1 as an example) and untreated (25 years old) testis by protein immunostaining of three germ cell markers. (E) Immunolocalization of germ cells and Sertoli cells in T-suppressed (Tf1 as an example) and untreated (25 years old) testis by staining for UTF1 and SOX9. See also Figure S6 and Table S4.

**Figure 6:**
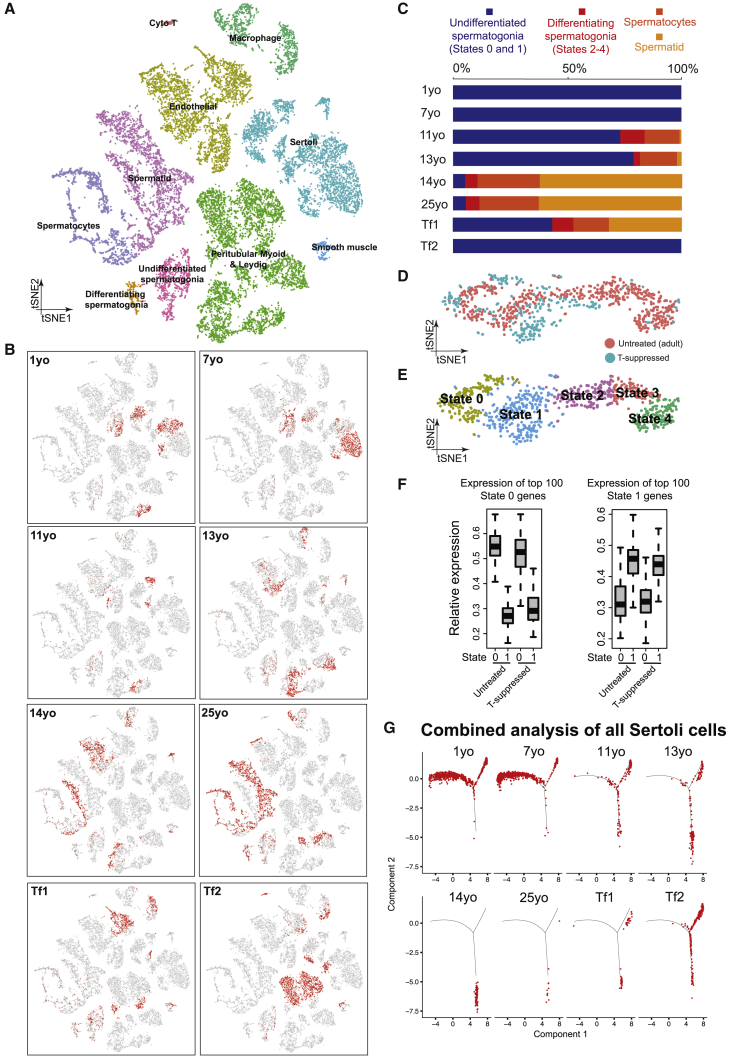
Testosterone Promotes Sertoli and Germ Cell Development *In Vivo* (A) tSNE and clustering analysis of combined single-cell transcriptome data from T-suppressed (Tf1 and Tf2) and untreated (from infancy through puberty to adult) testis, with cells colored based on their identities. See Figure S6D for the markers used to assign the cell identities. (B) Partitioning the combined tSNE analysis in Figure 6A based on the ages/donors of origin, with cells from each donor highlighted in red separately in different boxes. (C) Relative proportion of cells at different spermatogenic stages in untreated testes (different ages) or T-suppressed testes. (D and E) Comparison of spermatogonia from untreated adult (5 States as defined in Guo et al., 2018) and T-suppressed testes via tSNE analysis, with cells colored according to their T treatment (D) or spermatogonial states (E). (F) Expression levels of spermatogonial State 0 genes (left) or State 1 genes (right) in untreated or T suppressed. Neither State 0, nor State 1 cells from untreated and T-suppressed testes showed statistically significant differences in gene expression via Wilcoxon test (p value = 0.528 [left] and 0.065 [right]). (G) Comparison of Sertoli cells profile in all samples from T-suppressed (Tf1 and Tf2) and untreated (from infancy through puberty to adult) testes using focused pseudotime analysis (via Monocle). See also Figure S6.

Although previous data and our current analysis suggest a key role for AR and T in promoting testis development (both spermatogonia and niche cells) during puberty (Herbison, 2016, Koskenniemi et al., 2017), the molecular details involved are poorly characterized. Hence, to better define the functional role of T in the adult testis, we examined the expression profiles of testes from two adult transfemales who had been subjected to long-term reduction in T. One testis (Tf1) was from a T-suppressed 50-year-old transfemale, treated with spironolactone (a T antagonist) and estradiol for 19 months before testis removal during gender confirmation surgery. The other (Tf2) was obtained from a 26-year-old transfemale, treated with spironolactone and estradiol for 16 months before testis removal during gender confirmation surgery. In both cases, histological examination revealed major differences between the adult untreated and T-suppressed testes: macroscopically, the tubular architecture was disrupted and germ cell development was greatly impaired in the T-suppressed testes (Figure 5A). Here, scRNA-seq on isolated single cells from both T-suppressed testes was performed. For Tf1, our two technical replicates were highly similar (Figure S6A), yielding 2,161 single cells (after QC filtering). For Tf2, a single replicate yielding more than twice the cell numbers of Tf1 was analyzed. For both Tf1 and Tf2, tSNE and clustering analysis revealed the presence of the major testicular cell types as defined by clustering and annotation with key marker genes (Figures 5B and 5C). However, we noted that the relative proportion of cell types varied considerably in the two samples (Figure S6B). Thus, a larger study that examines and controls parameters (e.g., patient ages, treatment lengths, etc.) will be required to explain all differences. Nevertheless, our analyses reveal a set of consistent findings between the transfemale samples and replicates. For example, we observed a much lower proportion (in Tf1) or absence (in Tf2) of differentiating spermatogonia (e.g., *KIT*^+^), spermatocytes or spermatids, whereas in both transfemale samples spermatogonia expressing undifferentiated markers (e.g., *UTF1*^+^) were clearly present (Figures 5B–5E; Figure 6C; Figure S6B) and appeared relatively enriched in proportion when compared to untreated samples. In keeping with our scRNA-seq results, UTF1 protein expression was observed in both the T-suppressed and the untreated testes, while MKI67 and SYCP3 signals were both significantly reduced in the T-suppressed spermatogonia (Figure 5D). Interestingly, staining for the Sertoli cell marker SOX9 revealed a distorted localization pattern in the T-suppressed testes (Figure 5E), prompting further studies to better understand how T suppression affects Sertoli cells.

### T Promotes Germ Cell and Sertoli Cell Development *In Vivo*

To better understand the impact of T suppression on testicular cell development, we combined the scRNA-seq results from the two T-suppressed transfemales and those from all the 6 untreated males (from infancy through puberty into adulthood) and performed analysis (Figures 6A and 6B). Interestingly, spermatogonia derived from Tf1 and Tf2 cluster together with untreated spermatogonia (Figure 6B), suggesting that they do not transition to a new state but rather reside in state(s) present in untreated males. In keeping, examination of key markers that define spermatogonia states from the T-suppressed to the untreated testes revealed a higher proportion of undifferentiated spermatogonia (States 0 and 1) in the T-suppressed testes (Figure 6C). Further tSNE and clustering analysis of only spermatogonia demonstrated cells clustering based on “state” rather than “donor of origin” (Figures 6D and 6E). Results from two analyses further suggest that the undifferentiated spermatogonia from T-suppressed testes partition into State 0 and State 1 cells. First, we observed a clear separation of State 0 and State 1 cell clusters (Figures 6D and 6E); second, State 0 or 1 cells from either the untreated or T-suppressed testes expressed similar levels of State 0 or 1 specific markers (Figure 6F, based on a 100 gene classifier). Together, these results suggest that T has limited impact on transcriptional profiles of State 0–1 spermatogonia, though its suppression impairs progression to differentiation.

Additionally, we performed a combined analysis of Sertoli cells, and found that Sertoli cells from the two T-suppressed testes resembled more the 11- to 13-year-old samples than the 14-year-old or adult samples (Figure 6G). This is further supported by the upregulation of early Sertoli markers (including *AMH* and *HES1*), and downregulation of later Sertoli markers, including immune-related genes (*S100A6*, *ISG15*) (Figure S6E). Thus, T suppression in adults could lead to a partial reversal of Sertoli cell developmental state, mimicking aspects of the pubertal stage.

## Discussion

Puberty in human males involves major changes in testis weight and physiology, including development of niche cells to establish the seminiferous tubule, spermatogonial differentiation, and modulation of hormonal signaling—culminating in the establishment of steady spermatogenesis (Koskenniemi et al., 2017, Sharpe et al., 2003). To better understand this complex process at the molecular and genome-wide level, we generated the first overview of the pubertal transcriptional cell atlas at single-cell level, providing a foundational data resource coupled to multiple modes of analyses. To further facilitate data access and visualization, we have designed a website allowing browsing and query of expression patterns of genes of interest (https://humantestisatlas.shinyapps.io/humantestisatlas1/).

Regarding germ cell development, our prior dataset from adult human testes identified five cellular spermatogonial states (States 0–4) and provided evidence that States 0 and 1 correspond to the undifferentiated reserve germ cells (Guo et al., 2018). Notably, we observe a large increase (from 3% in the 7-year-old sample to ∼13% in the 11-year-old sample [Figure S1C]) in the proportion of spermatogonia (relative to the total testicular cells profiled). A similar increase has been previously documented in IHC studies examining the relative numbers of type A spermatogonia (Berensztein et al., 2002, Paniagua and Nistal, 1984)—which together suggest a proliferative phase involving expansion of States 0–1 spermatogonia concomitant with seminiferous tubule reorganization and development. Of note, our scRNA-seq and protein validation do not support a simple molecular basis to account for the two histologically distinct population of A_dark_ and A_pale_ spermatogonia noted previously via histology (Clermont, 1966). Our results are consistent with recent work (Jan et al., 2017), which did not identify significant transcriptional differences within laser-capture microdissected A_dark_ and A_pale_ spermatogonia, suggesting that additional proteomic and functional work is needed to understand possible differences. Notably, tubule morphogenesis (with a defined basal lamina and lumen) coincided with the first emergence of differentiated spermatogonia (States 2–4), without completion of gametogenesis, as very few spermatids were observed. Thus, a phase of spermatogonial expansion (involving States 0-1) and initial differentiation (involving States 2–4) precedes the ability to complete spermatogenesis. Furthermore, our results from juveniles and the transfemale testes strongly suggest that States 0 and 1 are indeed reserve (undifferentiated, quiescent, or slow-cycling) spermatogonia that persist from infancy to adulthood even in the absence/suppression of T. For example, in the T-suppressed testes, while spermatogenesis is severely impaired, we observed a population of spermatogonia with the same transcriptional profile as the State 0–1 population identified in untreated adult testes (Guo et al., 2018). This likely accounts for the ability of spermatogonia in some transfemales to resume gametogenesis when T suppression is halted (Kohn et al., 2017, Schneider et al., 2017).

Interestingly, whereas AR knockout or T-suppressed rodents display a germ cell developmental block only at/after meiosis (Walker, 2011, Yeh et al., 2002), our data correlate T upregulation with the emergence of differentiating spermatogonia, as well as a role in spermiogenesis. Notably, in macaque monkeys, treatment with GnRH inhibitor for 2 weeks reduced T and impaired development beyond the undifferentiated/self-renewing (A_dark_) spermatogonia but did not change the proportion of A_dark_ spermatogonia (O’Donnell et al., 2001, Weinbauer et al., 1998)—suggesting that both in macaques and humans spermatogonial differentiation may require T. Whether this effect is mediated by Sertoli cells, which harbor the receptor for T (AR), and/or if T directly regulate spermatogonia through an AR-independent pathway, remains unknown. Finally, despite our observations of the role of T, our pathway analyses suggest that additional ligands (e.g., Activin, NOTCH, etc.) may control human spermatogonial differentiation.

Our work reinforces prior work that the physiology of the postnatal testis is markedly different in mice versus humans. Pre-pubertal males display small testis cords lacking an apparent lamina or lumen, structures resembling those in fetal/early postnatal mouse testes, which in peri-pubertal stages progressively acquire a more tubular structure. Regarding the location of spermatogonia in these cords and nascent tubules, consistent with prior work (Paniagua and Nistal, 1984), we observe spermatogonia at both the basement membrane and within the cord/tubule in pre-pubertal samples (Figure S2F), which in peri-pubertal and subsequent stages become fully aligned with the basement membrane. Therefore, our data and analysis—together with prior work by others—suggest that the development and physiology of human spermatogonia from 1 year old through pre-puberty is very different in humans versus mice.

Regarding somatic cell development, our study reveals several insights. First, both spermatogonia and niche cell transcriptional profiles showed very little change between the 1- and 7-year-old samples—and then changed dramatically. For example, we observed (by transcriptional criteria) two distinct populations of Sertoli cells at infant-juvenile stages that differ in metabolic, mitochondrial, and cell-cycle properties—but lack major changes in transcription and chromatin factors—indicating that these states may not represent different developmental states but rather alternative physiological states. Surprisingly, these states appear to converge into a single maturing Sertoli population during puberty. Here, the expression of PDPN in the 11-year-old testis in only a subset of tubules suggests that Sertoli maturation as an asynchronous and gradual process. Future studies should focus on investigating the physiological, metabolic, and functional differences between Sertoli states, as well as the roles of the immune peptides and their regulation by T. Finally, our data suggest that anti-bacterial/viral peptides may be important for protection from infection associated with sexual activity after puberty.

Importantly, we identified a common precursor for Leydig and myoid cells in humans that expresses (at low and heterogeneous levels) markers normally associated with both mature Leydig and myoid cells. We also identified certain factors known in mouse to reside in fetal somatic precursors (e.g., *MAFB*) but did not observe *NESTIN* (a known marker of Leydig precursors in the fetal mouse) during the human stages we examined (data not shown) (Kumar and DeFalco, 2018). These human Leydig/myoid precursors persist from infancy until peri-puberty and then diverge and mature as the seminiferous tubules develop. Thus, our results reveal major differences between mice and humans regarding the markers for somatic cell development and the timing of their appearance—with Leydig and myoid lineages being specified during fetal stages in mice (Svingen and Koopman, 2013) but only at pre-/peri-pubertal stages in humans. We note that T-suppressed Leydig and myoid cells displayed altered transcriptomes that were not simply developmentally regressed patterns, prompting further investigation on the effect of T on the maintenance of Leydig and myoid cell functions. Moreover, the developmental timeline differences between humans and mice may account for differences in the composition of peritubular myoid cells (multi-layered in humans versus mono-layered in mice)—differences that could be further explored in non-human primate models.

Regarding limitations, our current study profiled and analyzed four testis samples spanning human puberty. Given that age differences in puberty onset exist in humans, and that puberty is a progressive process, future studies with additional samples may reveal additional details regarding developmental processes and transitions. Likewise, given the differences observed between the two transfemales profiled here, additional studies are needed to understand the origins of the variation observed. Furthermore, our analyses and interpretations focus primarily on transcriptional data supported by initial protein validation studies, and post-transcriptional mechanisms exist that will complement and refine our understanding of the molecular strategies and the complex interplay between the different cellular components of the juvenile testis.

The human testis is one of the few organs that defines most of its cell types, physiology, and function after birth. Our work provides major advances in defining the strategy and timing of human testis development. During pre-pubertal stages, the testis maintains a pool of undifferentiated and largely quiescent germline stem cells within an immature niche. During early puberty, maturation of the niche occurs first, involving the differentiation of a common progenitor to form mature Leydig and peritubular myoid cells, which contribute to defining the lamina-luminal architecture of the seminiferous tubule to promote signaling pathways—in conjunction with hormonal induction from the hypothalamic-pituitary-gonadal axis. Likewise, Sertoli cell populations develop and change their localization, and their transcriptional, metabolic, and signaling profiles. Proliferation of undifferentiated spermatogonia then commences to progressively populate the elongating tubules, followed by spermatogonial differentiation and the generation of the first meiotic cells, culminating in the steady generation of spermatids and mature sperm. Our data further strengthen the notion that establishment of spermatogenesis requires a complex interplay between multiple signaling pathways. These results should provide the foundation for building hypothesis-driven research that can be explored in primate models and will support improvement of *in vitro* cultures of human seminiferous tubules and spermatogonial maturation, to ultimately help guide therapeutic options for male infertility.

## STAR★Methods

### Key Resources Table

**Table:** 

REAGENT or RESOURCE	SOURCE	IDENTIFIER
**Antibodies**		

Mouse monoclonal anti-UTF1 Dilution: 1:100 - 1:1000	Millipore	cat# Mab4337 (5G10.2), RRID:AB_827541
Rat polyclonal anti-MKI67 Dilution: 1:100	Abcam	cat# ab15580, RRID:AB_443209
Rabbit monoclonal anti-MKI67 Dilution: 1:200	Abcam	cat# ab16667, RRID:AB_302459
Mouse polyclonal anti-ACTA2 Dilution: 1:400	Abcam	cat# ab5694, RRID:AB_2223021
Mouse monoclonal anti-ACTA2 Dilution: 1:300	Atlas Antibodies	cat# cab013531, RRID:AB_2677383
Mouse polyclonal anti-ACTA2 Dilution: 1:250	Sigma Aldrich	cat# A2547, RRID:AB_476701
Mouse monoclonal anti-AMH Dilution: 1:300	Santa Cruz	cat# sc-166752 RRID:AB_2289536
Mouse monoclonal anti-AR Dilution: 1:300	Atlas Antibodies	cat# CAB000001, RRID:AB_2685535
Rabbit monoclonal anti-AR Dilution: 1:250	Abcam	cat# ab133273, RRID:AB_11156085
Goat polyclonal anti-KIT Dilution: 1:25	R&D systems	cat# AF332, RRID:AB_355302
Rabbit polyclonal anti-INHA Dilution: 1:1000	Atlas Antibodies	cat# HPA019141, RRID:AB_1851789
Mouse monoclonal anti-LGALS3 Dilution: 1:500	Santa Cruz	cat# sc-32790, RRID:AB_627657
Rabbit polyclonal anti-MYH11 Dilution: 1:100	Atlas Antibodies	cat# HPA015310, RRID:AB_1854261
Rabbit polyclonal anti-S100A6 Dilution: 1:750	Atlas Antibodies	cat# HPA007575, RRID:AB_1079859
Rabbit polyclonal anti-SOX9 Dilution: 1:1000	Millipore	cat# AB5535, RRID:AB_2239761
Rabbit monoclonal anti-SOX9 Dilution: 1:300	Abcam	cat# ab185966, RRID:AB_2728660
Rabbit monoclonal anti-SYCP3 Dilution: 1:400	Abcam	cat# ab15093, RRID:AB_301639
Rabbit polyclonal anti-CYP11A1 Dilution: 1:250	Atlas Antibodies	cat# HPA016436, RRID:AB_1847423
Mouse polyclonal anti-PDPN Dilution: 1:500	Abcam	cat# ab77854, RRID:AB_1566117
Donkey anti-Mouse IgG (H+L) Highly Cross-Adsorbed Secondary Antibody, Alexa Fluor 488	Thermo Fisher Scientific	cat# A21202, RRID:AB_141607
Donkey anti-Goat IgG (H+L) Highly Cross-Absorbed Secondary Antibody, Alexa Fluor 555	Thermo Fisher Scientific	cat# A21432, RRID:AB_141788
Donkey anti-Rabbit IgG (H+L) Highly Cross-Adsorbed Secondary Antibody, Alexa Fluor 488	Thermo Fisher Scientific	cat# A31573, RRID:AB_141788
Donkey anti-Mouse IgG (H+L) Highly Cross-Adsorbed Secondary Antibody, Alexa Fluor 555	Thermo Fisher Scientific	cat# A31570, RRID:AB_2536180

**Biological Samples**		

Human testis samples for donors	DonorConnect	NA
Human testis samples from transfemale	Universityf of Utah Andrology laboratory	NA
Human testis samples for immunohistochemistry	MRC Centre for Reproductive Health, The Queen’s Medical Research Institute, The University of Edinburgh and Royal Hospital for Children and Young People	NA

**Deposited Data**		

Single cell RNA-seq for pre- and peri-pubertal human testes	This paper	GEO: GSE134144
Single cell RNA-seq for transfemale testes	This paper	GEO: GSE134144

**Software and Algorithms**		

Seurat (2.3.4)	Butler et al., 2018	https://satijalab.org/seurat/
Monocle (2.10.1)	Qiu et al., 2017	http://cole-trapnell-lab.github.io/monocle-release/
GO (David 6.7)	Huang et al., 2009	https://david-d.ncifcrf.gov
Cell Ranger (2.2.0)	NA	https://support.10xgenomics.com/single-cell-gene-expression/software/pipelines/latest/what-is-cell-ranger
Cluster 3.0	NA	http://bonsai.hgc.jp/∼mdehoon/software/cluster/software.htm
scran (1.6.5)	Lun et al., 2016	https://bioconductor.org/packages/3.7/bioc/vignettes/scran/inst/doc/scran.html

**Other**		

Single cell RNA-seq for infant and adult human testes	Guo et al., 2018	GEO: GSE120508
The Human Protein Atlas	Lindskog, 2016	http://www.proteinatlas.org

### Lead Contact and Materials Availability

Further information and requests for reagents should be directed to and will be fulfilled by the Lead Contact, Bradley R. Cairns (brad.cairns@hci.utah.edu). This study did not generate new unique reagents.

### Experimental Model and Subject Details

#### Human Testicular Tissues

Pre- and peri-pubertal human testicular samples were obtained from four healthy boys aged 7, 11, 13 and 14 years old, through the University of Utah Andrology laboratory and Connect Donor (formerly named Intermountain Donor Services). Those samples were removed from deceased individuals who consented to organ donation for transplantation and research.

Transfemale (testosterone-suppressed) samples were obtained through the University of Utah Andrology laboratory consented for research (IRB approved protocol #00075836).

Pre- and peri-pubertal tissues for orthogonal validation studies were obtained from males undergoing testicular tissue cryopreservation for fertility preservation with prior ethical approval from the South East Scotland Research Ethics Committee (Reference: 13SS/0145).

### Method Details

#### Sample Storage by Cryopreservation

Once collected, the pair of whole-testis was transported to the research laboratory on ice in saline or Hank’s Balanced Salt Solution (HBSS; GIBCO cat # 24020117) and processed within 1 hour of removal by surgery. Around 90% of each testis was divided into smaller portions (∼500 mg – 1g each) using scissors and directly transferred into cryovials (Corning cat # 403659) in DMEM medium (Life Technologies cat # 11995073) containing 10% DMSO (Sigma-Aldrich cat # D8779), 15% fetal bovine serum/FBS (GIBCO cat # 10082147) and cryopreserved in Mr. Frosty container (Thermo Fisher Scientific cat #5100-0001) at a controlled slow rate, and stored at −80°C for overnight. Cryovials were transferred to liquid nitrogen for long-term storage.

#### Sample Fixation for Immunostainings

Around 10% of the remaining testis tissues were placed in 1X PBS containing 4% paraformaldehyde/PFA (Thermo Fisher Scientific cat # 28908) and incubated overnight at 4C with agitation on a rotor (60 rpm). Fixed samples were then washed with three times in cold PBS, and stored in PBS at 4C until immunostaining processing.

#### Human Testis Sample Preparation for Single Cell RNA Sequencing

For each single-cell sequencing experiment, 1 or 2 cryovials were thawed quickly. Tissues were then washed twice with PBS, and subject to digestion as described previously (Guo et al., 2018). For pubertal samples, tissues were washed twice in 1 x PBS, and minced into small pieces for better digestion outcome. Tissues were then treated with trypsin/ethylenediaminetetraacetic acid (EDTA; Invitrogen cat # 25300054) for 20-25 min and collagenase type IV (Sigma Aldrich cat # C5138-500MG) at 37°C. For the transfemale samples, tissues were digested following the standard two step enzymatic isolation protocol as described in (Guo et al., 2018). The digestion was then stopped by adding 10% FBS (GIBCO cat # 10082147). Single testicular cells were obtained by filtering through 70 μm (Fisher Scientific cat # 08-771-2) and 40 μm (Fisher Scientific cat # 08-771-1) strainers. The cells were pelleted by centrifugation at 600 g for 15 min, and wash with PBS twice. Cell number was counted using hemocytometer, and the cells were then resuspended in PBS + 0.4% BSA (Thermo Fisher Scientific cat # AM2616) at a concentration of ∼1,000 cells/uL ready for single-cell sequencing.

#### Single Cell RNA-seq Performance, Library Preparation and Sequencing

Here, we aimed to capture ∼4,000-5,000 cells. Briefly, cells were diluted following manufacturer’s instructions, and 33.8 uL of total mixed buffer together with cells were loaded into 10x Chromium Controller using the Chromium Single Cell 3′ v2 reagents. The sequencing libraries were prepared following the manufacturer’s instructions, using 13 cycles of cDNA amplification, followed by an imput of ∼100 ng of cDNA for library amplification using 12 cycles. The resulting libraries were then sequenced on a 2 X 100 cycle paired-end run on an Illumina HiSeq 2500 or Novaseq 6000 instruments.

#### Processing of Single Cell RNA-seq Data

Raw data were demultiplexed using mkfastq application (Cell Ranger v2.2.0) to make Fastq files. Fastq files were then run with count application (Cell Ranger v2.2.0) using default settings, which performs alignment (using STAR aligner), filtering and UMI counting. The UMI count tables were used for further analysis.

#### Immunostaining of Testicular Tissues

The immunofluorescence (IF) stainings were performed on 5μm formalin-fixed paraffin embedded (FFPE) or cryopreserved sections from portions of the collected testicular samples following deparaffinisation, rehydratation and heat-mediated antigen retrieval in 10mM sodium citrate buffer solution (pH 6). After treatment with Superblock (PBS) Blocking Buffer (Thermo Fisher Scientific, cat# 37515) for 30 mins, individual sections were incubated overnight at 4°C with a mix of diluted antibodies (for antibodies details and dilutions, see the Key Resources Table). Antigen detection was conducted using the appropriate combination of Alexa Fluor 488 and 555 secondary antibodies (all 1:500; Thermo Fisher Scientific, cat#A21202, cat#A21432, cat#A31570, cat#A21206, respectively) for 2 hr at room temperature in the dark. All primary/secondary antibodies were diluted in SignalBoost Immunoreaction Enhancer Kit (Calbiochem, cat#407207-1KIT). After three washes in PBS, sections were incubated with DAPI (4’,6-Diamidine-2-phenylindole dihydrochloride) (Roche, cat#10236276001) to facilitate nuclear visualization (dilution 1:2000). Specificity of the antibody staining was confirmed using the same protocol but with omission of primary antibodies. Following multiple washes in PBS, slides were mounted using Vectashield mounting medium for fluorescence (Vector Laboratories, Inc., Burlingame, CA, cat#H-1000). Images were obtained under 25x objective (LD LCI PA 25x/0.8 DIC WD = 0.57 mm Imm Corr (UV)VIS-IR (Oil-Immersion) with a Zeiss LSM 780 Upright Multi-Photon Confocal Microscope and analyzed using ImageJ software (NIH, Bethesda, MD, USA).

#### Immunohistochemistry staining

For validation using the four pubertal samples, immunohistochemistry on 5 μm sections from FFPE or cryopreserved testis samples was performed using the Rabbit specific HRP/DAB (ABC) Detection IHC Kit (Abcam, cat# 236466). Briefly, after deparaffinisation, rehydration and heat-mediated antigen retrieval in 10mM sodium citrate buffer solution (pH 6), sections were blocked with Protein block and incubated overnight at 4°C with a variety of different antibodies in 1x TBS. Subsequently, sections were incubated with HRP conjugate before chromogenic detection using DAB (3,3′Diaminobenzidine). Nuclei were counterstained with hematoxylin (Poly Scientific, cat#s212A), slides mounted in Aquatex (Merck, cat#108562) and images were acquired using the Hamamatsu NanoZoomer slide scanner and the NDP.view 2 software (Hamamatsu Photonics).

For validation in additional human testis tissue samples, immunohistochemistry was performed on Bouin’s fixed, paraffin-embedded tissue sections of approximately 5μm thickness. The sections were deparaffinized with xylene and rehydrated in ethanol using a reducing concentration from 100% to 70% (20secs each). Between each step sections were washed in TBS. If required, heat-mediated antigen retrieval in 10mM citrate buffer, pH6 was performed in a pressure cooker. Sections were cooled in TBS and endogenous peroxidase activity was blocked in 3% (v/v) hydrogen peroxide (VWR International) in methanol (Fisher Chemicals) for 30mins. Sections were blocked in Normal Horse Serum (Diagnostics Scotland) diluted (1:5) in TBS with 5% Bovine Serum Albumin (Sigma Aldrich). Sections were incubated in primary antibody diluted in blocking serum overnight in a humidified chamber at 4°C. The following day, sections were incubated with the relevant Impress HRP secondary antibody at room temperature for 30mins. Visualization of staining was performed using DAB (1drop/ml) diluted in buffer. Sections were counterstained with Haematoxylin for 2.5mins, Scott’s tap water for 30 s and dehydrated by gradual introduction to ethanol from 70% to 100% (20secs each) and Xylene (2x 5mins). Sections were mounted using Pertex (CellPath). Brighfield images were captured using the ZEISS Axio Scan.Z1 at 20x magnification.

#### H&E staining

For hematoxylin and eosin (H&E) staining, deparaffinized and rehydrated 5 μm sections were incubated in hematoxylin for 3 min and rinsed with running tap water for 5 min. Afterward, the sections were dipped in acid alcohol (0,5% v/v hydrochloric acid in 70% ethanol), washed with distilled H2O, and incubated in eosin (Poly Scientific, cat#176) for 30 s. The sections were dehydrated before mounting with Histomount (National Diagnostics, cat# 12954910).

Johnsen scores are the standard quantitative/clinical histological grading system utilized by testis pathologists, which assesses the degree of spermatogenic maturation in individual tubular cross-sections, ranging between 1 to 10, with 1 representing complete azoospermia, and 10 representing full spermatogenesis(Johnsen, 1970).

#### Cell counting

For quantification of UTF and/or KIT expressing cells, single-positive and double-positive cells were counted in cross-sections of seminiferous tubules. The number of positive cells per cross-section were normalized to the total number of cells located in the periphery of the seminiferous tubule. The bars represent means + SD of independent tubules. Data were analyzed using unpaired two-sided Student’s t test. p < 0.05 was considered statistically significant.

### Quantification and Statistical Analysis

The Seurat program (https://satijalab.org/seurat/, R package, v.2.3.4) was used as a first analytical package. To start with, UMI count tables from both replicates from all four juvenile donors were loaded into R using Read10X function, and Seurat objects were built from each experiment. Each experiment was filtered and normalized with default settings. Specifically, cells were retained only if they contained > 500 expressed genes, and had < 25% reads mapped to mitochondrial genome. We first run t-SNE and clustering analysis on each replicate, which resulted in similar t-SNE map. Data matrices from different donors and replicates were then combined with the previously published infant and adult data (Guo et al., 2018). Next, cells were normalized to the total UMI read counts, as instructed in the tutorial (http://satijalab.org/seurat/). t-SNE and clustering analyses were performed on the combined data using the top 6,000 highly variable genes and 1-30 PCs, which showed the most significant p values.

Detailed pseudotime for different cell types were performed using the Monocle package (v2.10.1) following the default settings. After pseudotime coordinates/order were determined, gene clustering analysis was performed to establish the accuracy of pseudotime ordering. Here, cells (in columns) were ordered by their pseudotime, and genes (in rows) were clustered by k-means clustering using Cluster 3.0. Different k-mean numbers were performed to reach the optimal clustering number. Cell cycle analysis was performed using scran program (https://bioconductor.org/packages/3.7/bioc/vignettes/scran/inst/doc/scran.html, R Package; v1.6.5).

### Data and Code Availability

All software tools can be found online (see Key Resources Table). The accession number for all sequencing data reported in this paper is GEO: GSE134144.

## Supplemental Information

Document S1. Figures S1–S6

Table S1. Summary of Juvenile Single-Cell Dataset and Clustering Information, Related to Figure 1

Table S2. Genes that Show Differential Expression in Two Immature Sertoli States, Related to Figure 3

Table S3. Genes that Show Differential Expression along Leydig-Myoid Cell Development, Related to Figure 4

Table S4. Summary of Transfemale Single-Cell Dataset, Related to Figure 5

Document S2. Article plus Supplemental Information
