# Supplementary material for: The Dynamic Transcriptional Cell Atlas of Testis Development during Human Puberty
Source: Cell Stem Cell. Author manuscript; Available in PMC 2022 May 2. (PMC7298616; doi:10.1016/j.stem.2019.12.005)
Supplement: 2 [file NIHMS1569464-supplement-2.pdf]

Supplemental Figures

Fig. S1

A

	Total reads	Mean reads/cell	Median gene/cell	Detected gene #	Captured cell #	Sequencing Saturation	Correlation
7yo Rep 1	210 M	203,705	1,612	20,932	1,030	93.4%	0.980
7yo Rep 2	291 M	310,094	1,644	20,868	938	95.5%	
11yo Rep 1	288 M	114,236	906	22,181	2,524	86.1%	0.964
11yo Rep 2	292 M	176,674	1,165	22,995	1,652	88.1%	
13yo Rep 1	263 M	125,116	1,482	24,514	2,104	84.5%	0.985
13yo Rep 2	280 M	143,635	1,684	24,510	1,947	84.8%	
14yo Rep 1	288 M	213,918	2,083	26,935	1,116	88.0%	0.994
14yo Rep 2	285 M	207,187	1,944	26,756	1,132	87.4%	

B

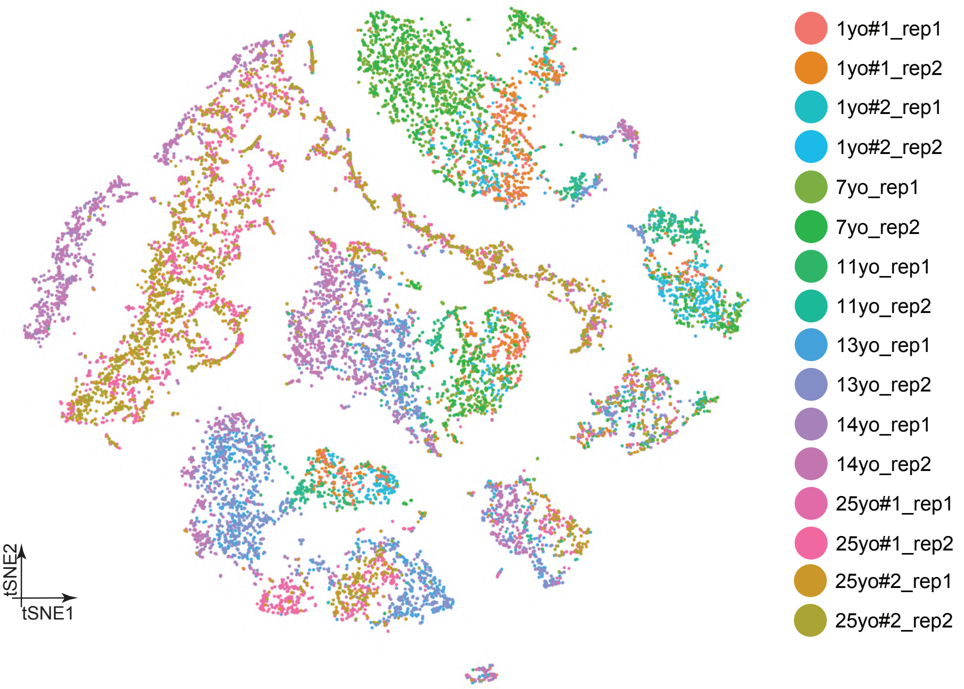

C

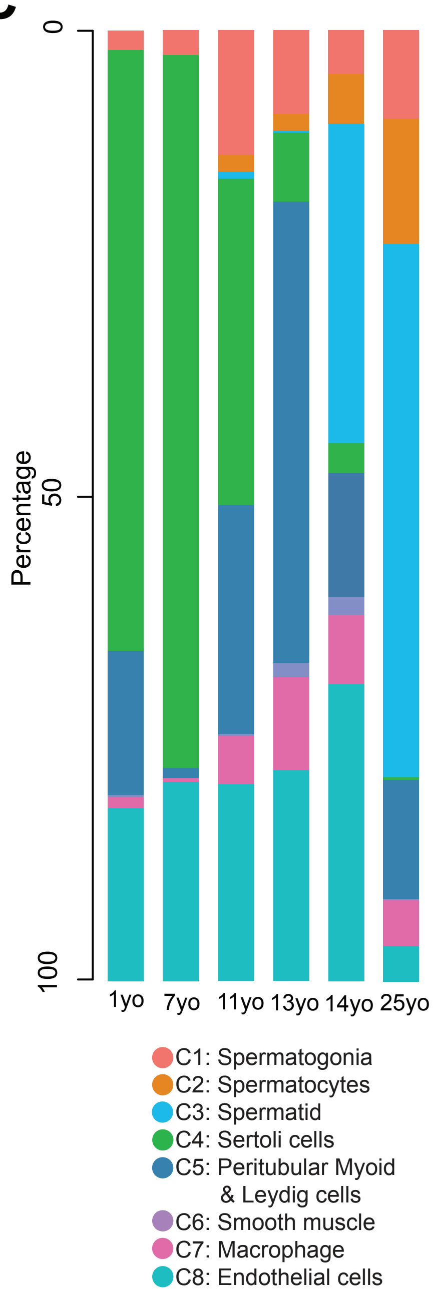

D

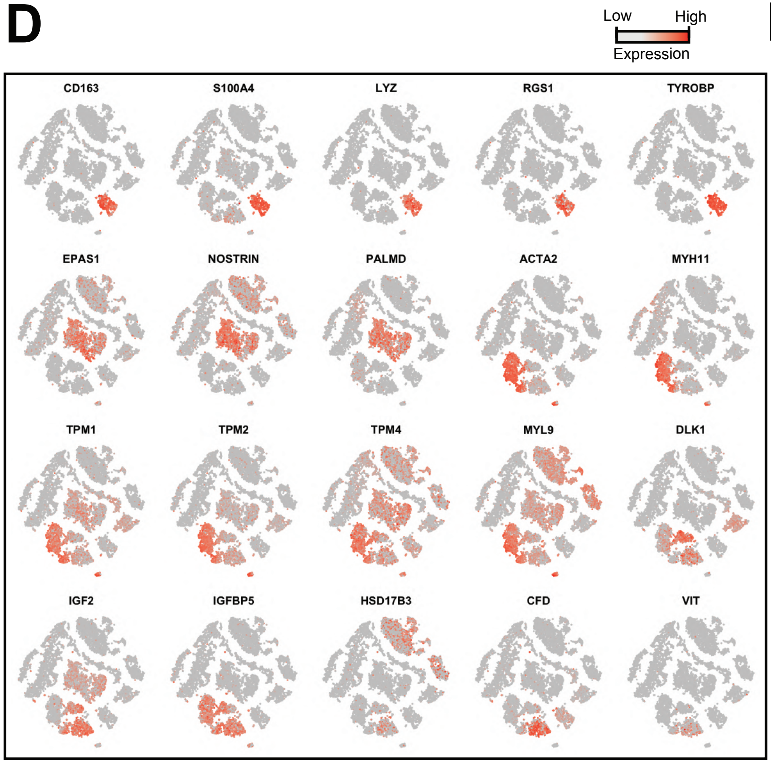

E

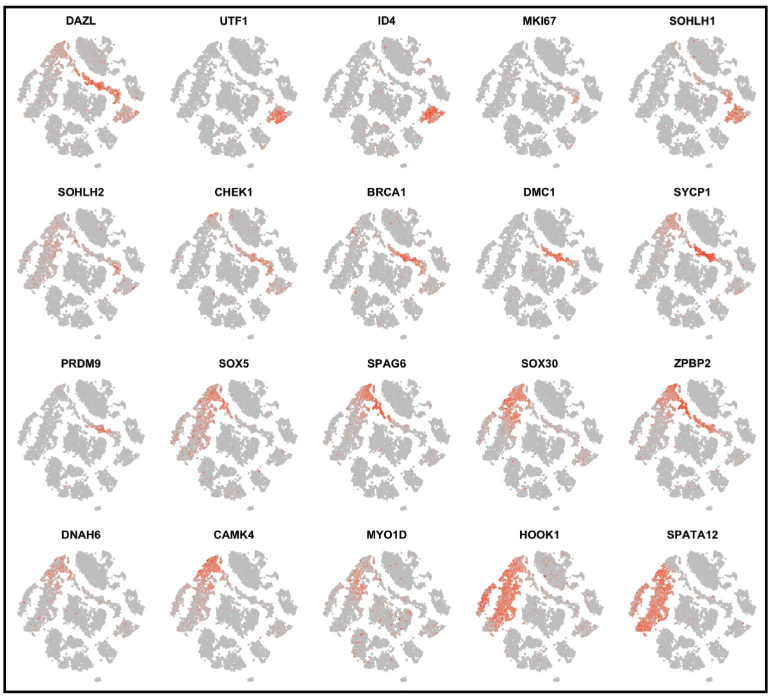

Figure S1. Quality control metrics of single-cell RNA-seq datasets
(A) Sequencing metrics of eight datasets (four juvenile samples; two technical replicates each sample) generated.
(B) tSNE plot of single-cell transcriptome with cells colored according to their donor of origin and replicate.
(C) Bar graph showing the relative proportion of different cell types/clusters for each sample/age.
(D) Gene expression patterns of additional markers of niche/somatic cells, projected onto the tSNE plot of Figure 1C.
(E) Gene expression pattern of additional markers in germ cells. Genes have been organized to mark more differentiated germ cell populations as they move from the top to the bottom rows.
Related to Figure 1.

Fig. S2

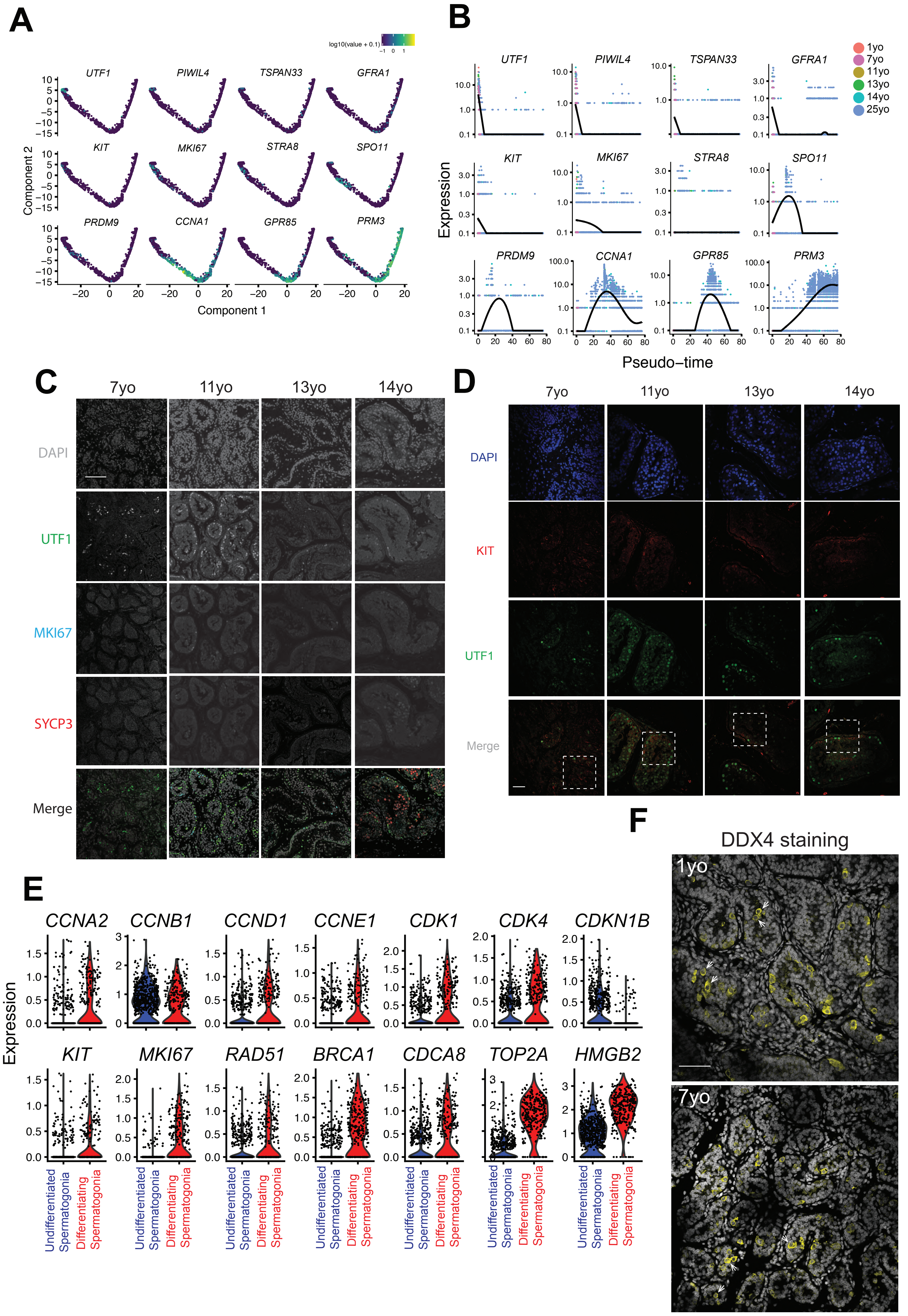
A
B
C
D
E
F
UTF1
PIWIL4
TSPAN33
GFRA1
KIT
MKI67
STRA8
SPO11
PRDM9
CCNA1
GPR85
PRM3
Component 1
Component 2
log10(value + 0.1)
1yo
7yo
11yo
13yo
14yo
25yo
Expression
Pseudo−time
7yo
11yo
13yo
14yo
DAPI
UTF1
MKI67
SYCP3
Merge
KIT
CCNA2
CCNB1
CCND1
CCNE1
CDK1
CDK4
CDKN1B
RAD51
BRCA1
CDCA8
TOP2A
HMGB2
Undifferentiated Spermatogonia
Differentiating Spermatogonia
DDX4 staining
1yo
7yo

Figure S2. Distinct phases of spermatogonial proliferation and differentiation during human puberty

(A) Expression patterns of representative markers projected onto the Monocle plot of Figure 2C. Key represents gene expression levels.

(B) Expression levels of representative dynamic genes during germ cell development, as predicted by pseudotime trajectory.

(C) Protein immunostaining of the samples analyzed (7-14yo) using three different germ cell markers: UTF1 is an undifferentiated spermatogonia marker (State 0-1), MKI67 expression overlaps with proliferating/differentiating spermatogonia, and SYCP3 is a meiotic marker. White bar represents = 100um.

(D) Low magnification of the FFPE section in Fig 2F. The boxed regions represent the part of the area shown at the higher magnification on Fig 2F. White bar represents = 20um.

(E) Violin plots of representative cell cycle related genes in undifferentiated and differentiating spermatogonia.

(F) IF staining for DDX4 in the pre-pubertal (1 and 7yo) testes. Although most germ cells (yellow) localize at the basement membrane, some germ cells do not (as indicated by white arrows). White bar represents = 100um.

Related to Figure 2.

Fig. S3

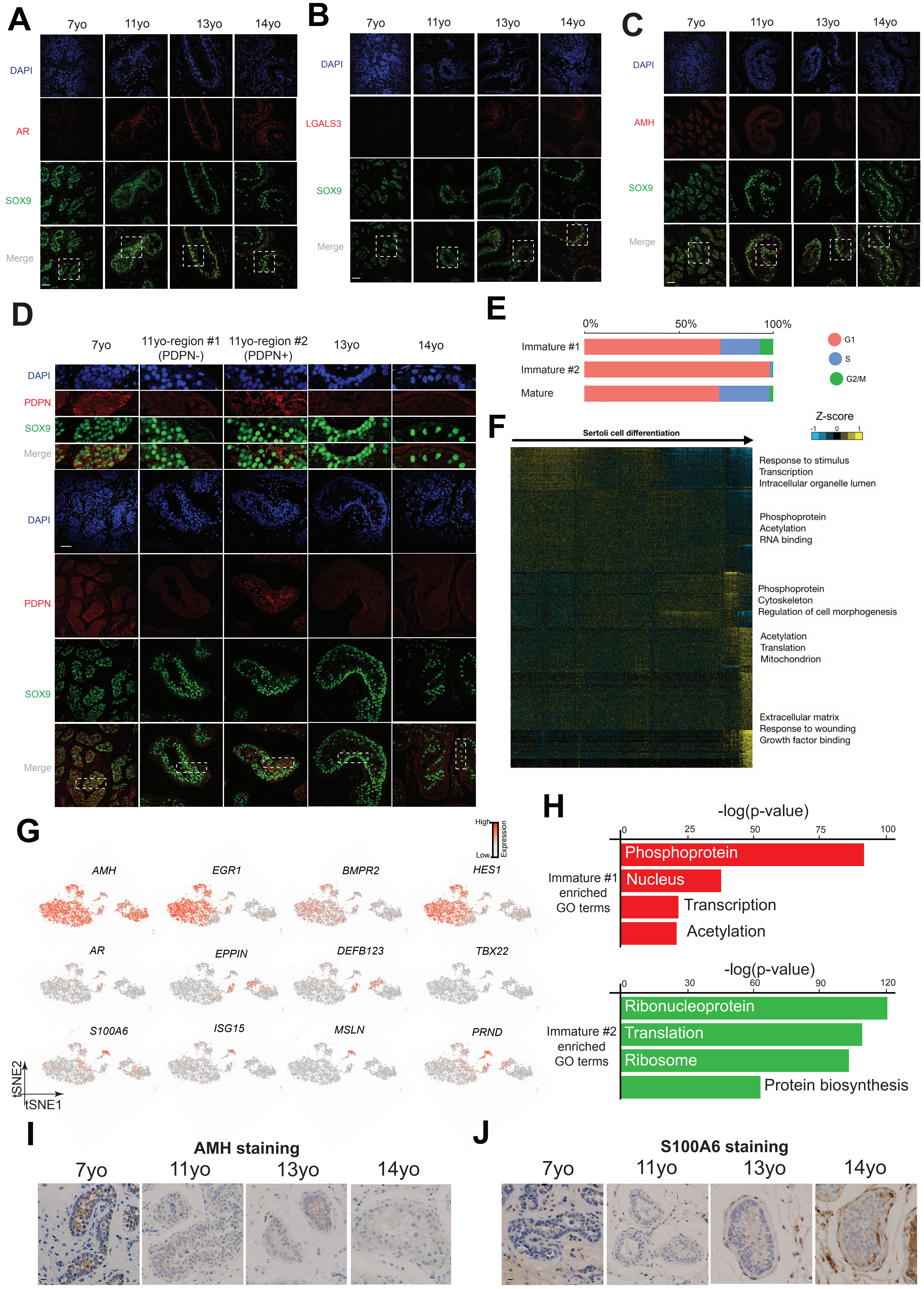

A
7yo
11yo
13yo
14yo
DAPI
AR
SOX9
Merge
B
LGALS3
C
AMH
D
11yo-region #1 (PDPN-)
11yo-region #2 (PDPN+)
PDPN
E
0%
50%
100%
Immature #1
Immature #2
Mature
G1
S
G2/M
F
Sertoli cell differentiation
Z-score
-1
0
1
Response to stimulus
Transcription
Intracellular organelle lumen
Phosphoprotein
Acetylation
RNA binding
Phosphoprotein
Cytoskeleton
Regulation of cell morphogenesis
Acetylation
Translation
Mitochondrion
Extracellular matrix
Response to wounding
Growth factor binding
G
High
Low
Expression
AMH
EGR1
BMPR2
HES1
AR
EPPIN
DEFB123
TBX22
S100A6
ISG15
MSLN
PRND
tSNE2
tSNE1
H
-log(p-value)
0
25
50
75
100
Immature #1 enriched GO terms
Phosphoprotein
Nucleus
Transcription
Acetylation
-log(p-value)
0
30
60
90
120
Immature #2 enriched GO terms
Ribonucleoprotein
Translation
Ribosome
Protein biosynthesis
I
AMH staining
J
S100A6 staining

Figure S3. Sertoli expansion and maturation during puberty
(A) Low magnification of the FFPE section in Fig 3H. The boxed regions represent the part of the area shown at the higher magnification on Fig 3H. White bar represents = 20um.
(B) Low magnification of the FFPE section in Fig 3I. The boxed regions represent the part of the area shown at the higher magnification on Fig 3I
(C) Low magnification of the FFPE section in Fig 3J. The boxed regions represent the part of the area shown at the higher magnification on Fig 3J.
(D) Immunofluorescent co-staining for SOX9 and PDPN at different ages (7-14yo) supports the gradual maturation of Sertoli cells over time observed by pseudotime trajectory.
(E) Deconvolution of the cell cycle phases (G1/S/G2) for the distinct populations of Sertoli cell States.
(F) K-means clustering of genes exhibiting differential expression (n=1000) during Sertoli cell differentiation/maturation. Note: each row represents a gene, and each column represents a single cell, with columns/cells placed in pseudo-developmental order. Differential gene expression levels utilize a Z score, which represents the variance from the mean, as defined on the color key in the right top corner, with associated GO terms (using DAVID v6.7) described on the right of the corresponding gene clusters.
(G) Expression patterns of representative markers projected onto the tSNE plot in Figure 3B.
(H) GO analysis of differential expressed programs that are associated with Immature Sertoli #1 (top) or Immature Sertoli #2 (bottom).
(I) Immunohistochemistry of the early Sertoli marker AMH at different ages (7-14yo). Black bar represents 20um.
(J) Immunohistochemistry of the later Sertoli marker S100A6 at different ages (7-14yo). Black bar represents 20um.
Related to Figure 3.

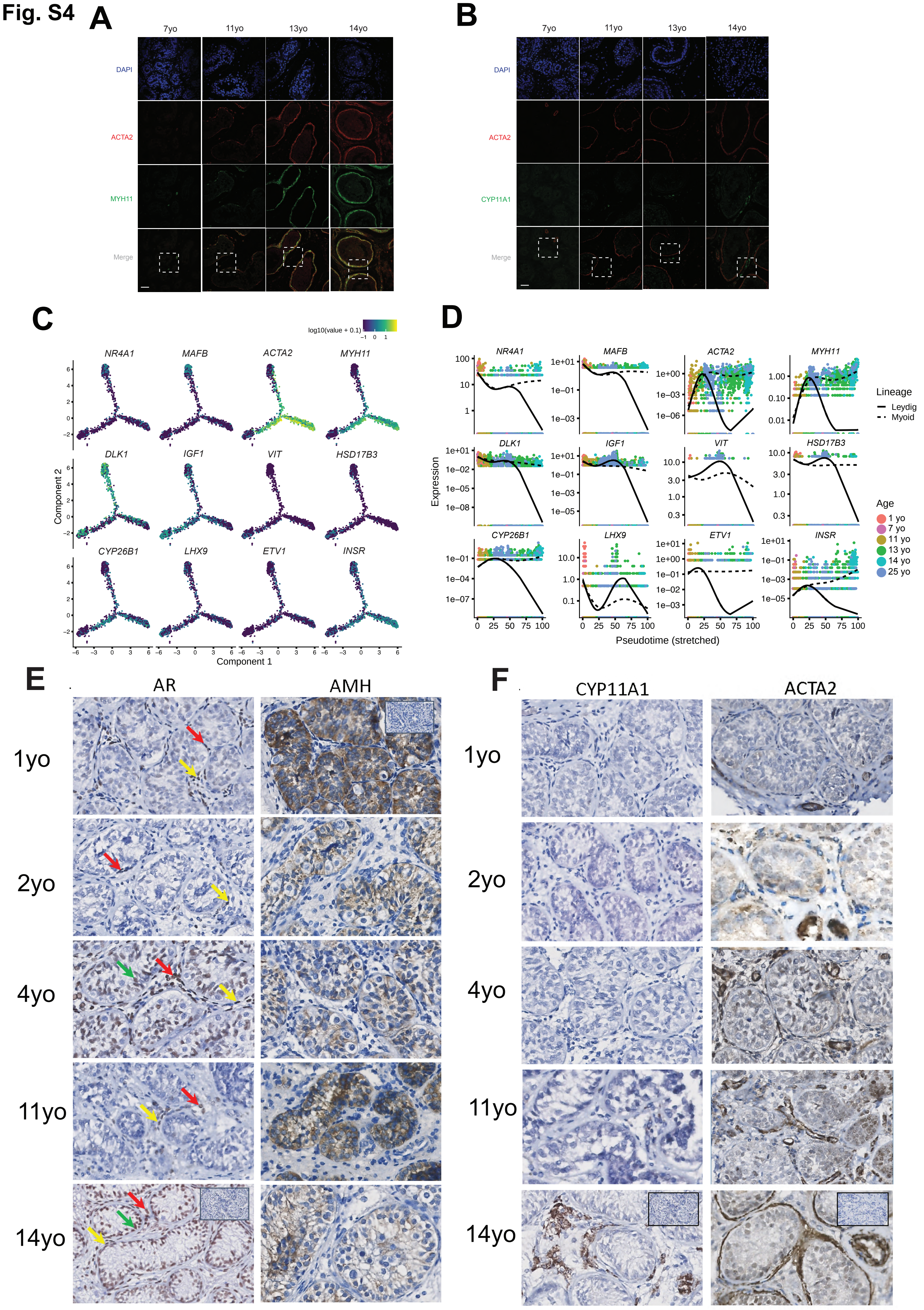

Fig. S4
A
7yo
11yo
13yo
14yo
DAPI
ACTA2
MYH11
Merge
B
7yo
11yo
13yo
14yo
DAPI
ACTA2
CYP11A1
Merge
C
log10(value + 0.1)
NR4A1
MAFB
ACTA2
MYH11
DLK1
IGF1
VIT
HSD17B3
CYP26B1
LHX9
ETV1
INSR
Component 2
Component 1
D
NR4A1
MAFB
ACTA2
MYH11
DLK1
IGF1
VIT
HSD17B3
CYP26B1
LHX9
ETV1
INSR
Expression
Pseudotime (stretched)
Lineage
Leydig
Myoid
Age
1 yo
7 yo
11 yo
13 yo
14 yo
25 yo
E
AR
AMH
1yo
2yo
4yo
11yo
14yo
F
CYP11A1
ACTA2
1yo
2yo
4yo
11yo
14yo

Figure S4. Leydig and myoid cells orgniate from a common precursor at prepuberty
(A) Low magnification of the FFPE section in Fig 4G. The boxed regions represent the part of the area shown at the higher magnification on Fig 4G. White bar represents = 20um.
(B) Low magnification of the FFPE section in Fig 4H. The boxed regions represent the part of the area shown at the higher magnification on Fig 4H.
(C) Expression patterns of representative markers projected onto the Monocle plot in Figure 4C
(D) Expression patterns of representative dynamic genes along Leydig (solid line) or myoid (dash line) lineage specification, as predicted by pseudotime.
(**E-F**) Orthogonal validation of genomics findings via additional juvenile testis samples. Immunohistochemistry staining of Sertoli cell markers (E) and Leydig/myoid markers (F) using additional juvenile samples. Yellow arrows: peritubular myoid cells; green arrows: Sertoli cells; red arrows: Interstitial (Leydig) cells.
Related to Figures 3 and 4.

Fig. S5

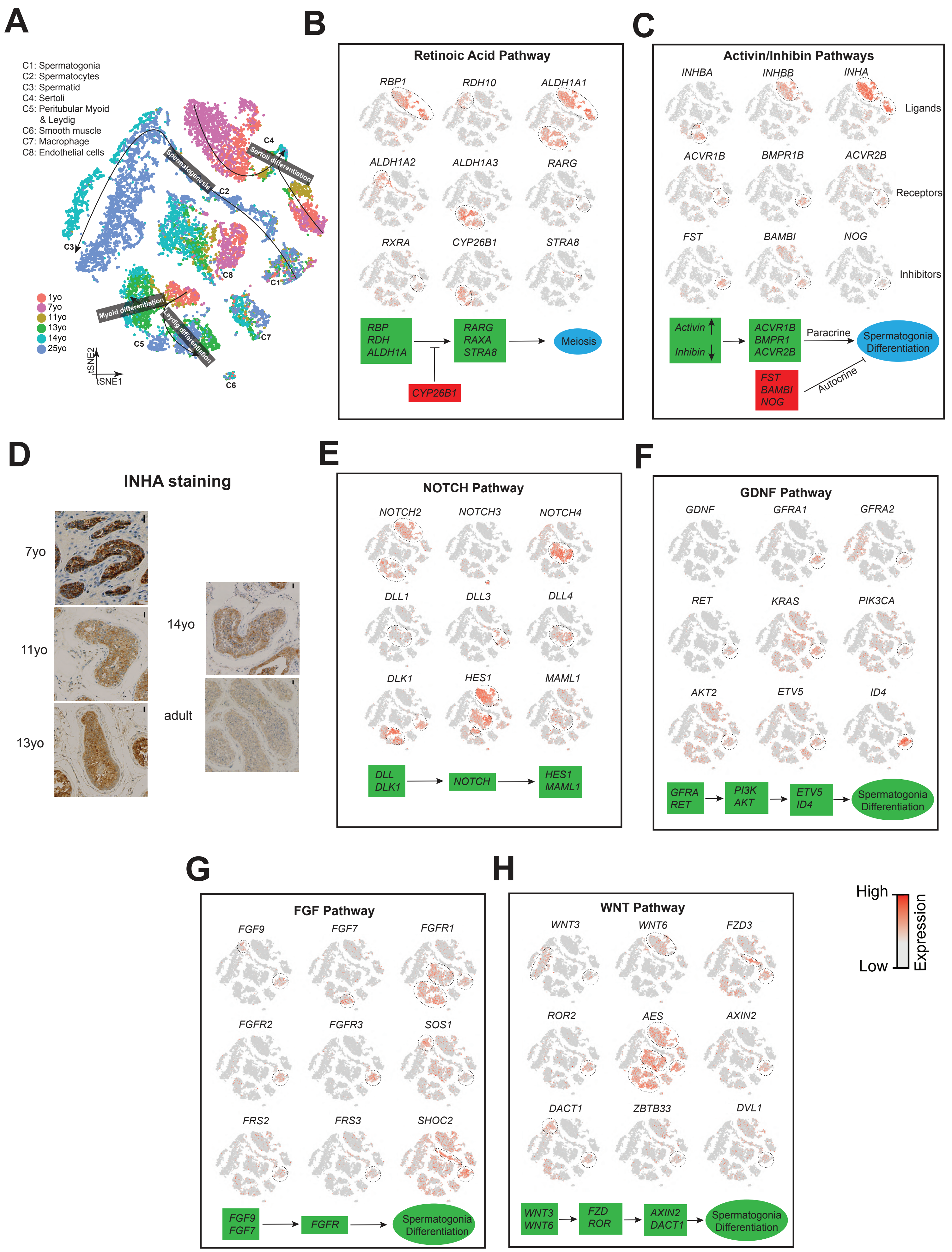
A
C1: Spermatogonia
C2: Spermatocytes
C3: Spermatid
C4: Sertoli
C5: Peritubular Myoid & Leydig
C6: Smooth muscle
C7: Macrophage
C8: Endothelial cells
Spermatogenesis
Sertoli differentiation
Myoid differentiation
Leydig differentiation
1yo
7yo
11yo
13yo
14yo
25yo
tSNE1
tSNE2
B
Retinoic Acid Pathway
RBP1
RDH10
ALDH1A1
ALDH1A2
ALDH1A3
RARG
RXRA
CYP26B1
STRA8
RBP
RDH
ALDH1A
RARG
RAXA
STRA8
Meiosis
CYP26B1
C
Activin/Inhibin Pathways
INHBA
INHBB
INHA
Ligands
ACVR1B
BMPR1B
ACVR2B
Receptors
FST
BAMBI
NOG
Inhibitors
Activin
Inhibin
ACVR1B
BMPR1
ACVR2B
Paracrine
Spermatogonia Differentiation
FST
BAMBI
NOG
Autocrine
D
INHA staining
7yo
11yo
13yo
14yo
adult
E
NOTCH Pathway
NOTCH2
NOTCH3
NOTCH4
DLL1
DLL3
DLL4
DLK1
HES1
MAML1
DLL
DLK1
NOTCH
HES1
MAML1
F
GDNF Pathway
GDNF
GFRA1
GFRA2
RET
KRAS
PIK3CA
AKT2
ETV5
ID4
GFRA
RET
PI3K
AKT
ETV5
ID4
Spermatogonia Differentiation
G
FGF Pathway
FGF9
FGF7
FGFR1
FGFR2
FGFR3
SOS1
FRS2
FRS3
SHOC2
FGF9
FGF7
FGFR
Spermatogonia Differentiation
H
WNT Pathway
WNT3
WNT6
FZD3
ROR2
AES
AXIN2
DACT1
ZBTB33
DVL1
WNT3
WNT6
FZD
ROR
AXIN2
DACT1
Spermatogonia Differentiation
High
Low
Expression

Figure S5. Overview of the key signaling pathways regulating testis development
(A) Summary of the tSNE plot of the combined single-cell transcriptome dataset, with the solid curve lines (with arrows) depicting the inferred developmental trajectories of the different cell clusters.
(B) Relative expression levels of representative genes from the retinoic acid (RA) pathway projected onto the tSNE plot from Figure S5A. Stage-specific expression is highlighted by dotted circles.
(C) Relative expression levels of representative genes from the Activin/Inhibin pathway projected onto the tSNE plot from Figure S5A. Stage-specific expression is highlighted by dotted circles.
(D) Immunohistochemistry on the analyzed samples shows that INHA expression (brown) decreases as puberty proceeds. Scale bar: 20um.
(E) Relative expression levels of representative genes within the NOTCH pathway projected onto the tSNE plot from Figure S5A. Stage-specific expression is highlighted by dotted circles.
(F) Relative expression levels of representative genes within the GDNF pathway projected onto the tSNE plot from Figure S5A. Stage-specific expression is highlighted by dotted circles.
(G) Relative expression levels of representative genes within the FGF pathway projected onto the tSNE plot from Figure S5A. Stage-specific expression is highlighted by dotted circles.
(H) Relative expression levels of representative genes within the WNT pathway projected onto the tSNE plot from Figure S5A. Stage-specific expression is highlighted by dotted circles.
Related to Figures 3 and 4.

Fig. S6

A

	Total reads	Mean reads/cell	Median gene/cell	Detected gene #	Captured cell #	Sequencing Saturation	Correlation
Transfemale #1 Rep 1	365 M	295,338	1,823	26,295	1,235	92.1%	0.978
Transfemale #1 Rep 2	434 M	287,884	1,877	26,412	1,509	93.2%	
Transfemale #2	234 M	58,884	1,282	22,683	4,036	83.1%	NA

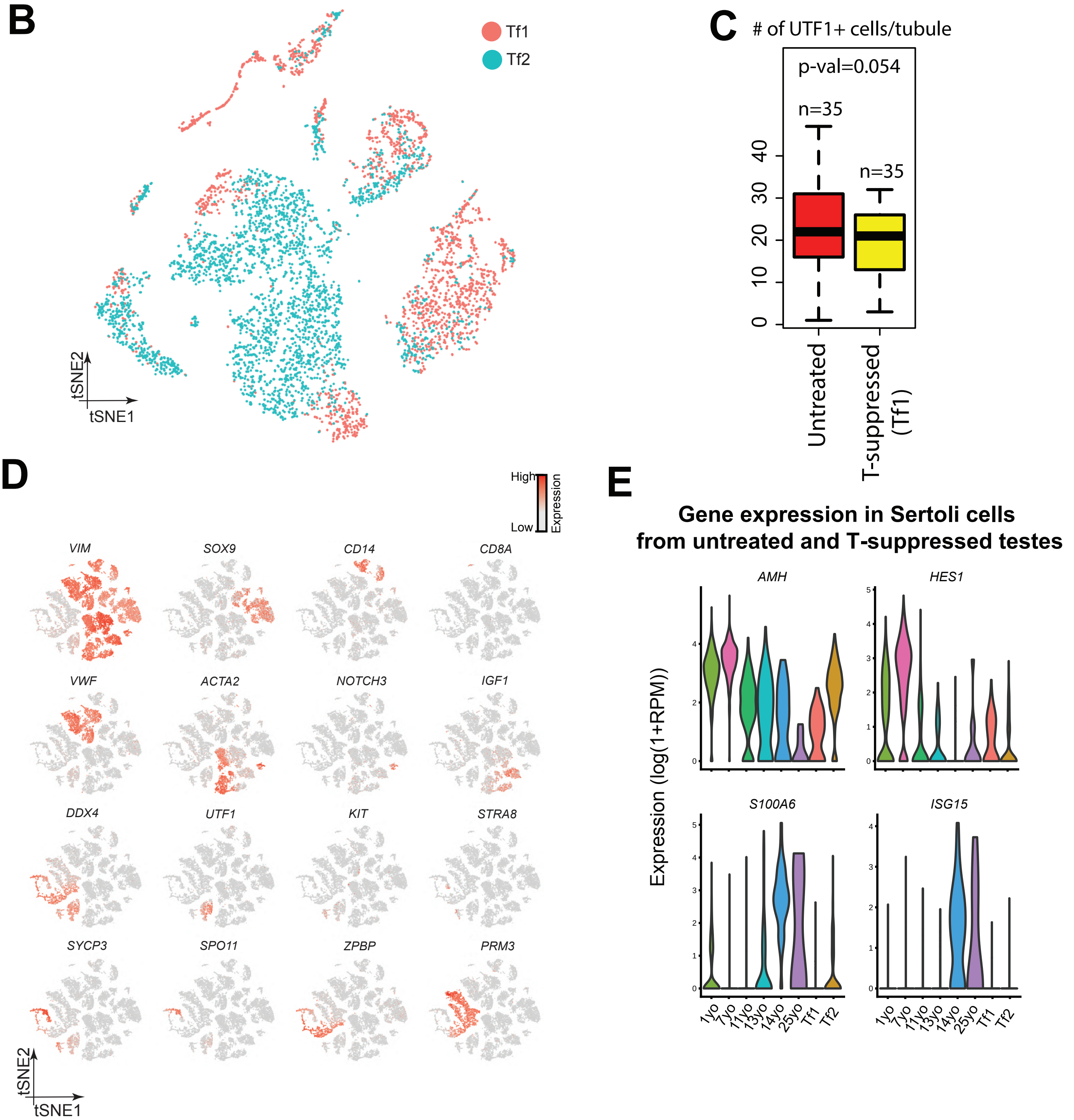

Figure S6. Single cell transcriptome profiling of testis from T-suppressed transfemales
(A) Sequencing metrics of three datasets generated.
(B) tSNE plot of single-cell transcriptome with cells colored based on their donor of origin.
(C) Quantification of number of UTF1+ quiescent spermatogonia (State 0-1) in untreated (25yo) and T-suppressed (Tf1) testis. No significant difference between the samples was observed ((Student t-test); p-value = 0.054).
(D) Expression patterns of selected markers projected onto the tSNE plot of Figure 6A (combined analysis of all untreated and T-suppressed samples). Top two rows represent somatic/niche cell markers; bottom two rows are representative germ cell markers.
(E) Violin plots showing the expression levels of representative Sertoli markers in T-suppressed and untreated at different ages in Sertoli cell populations.
Related to Figures 5 and 6.

Table S1: Summary of juvenile single-cell dataset and clustering information.
Related to Figure 1.

Table S2: Genes that show differential expression in two immature Sertoli States.
Related to Figure 3.

Table S3: Genes that show differential expression along Leydig-myoid cell development.
Related to Figure 4.

Table S4: Summary of transfemale single-cell dataset.
Related to Figure 5.
